# Mapping single-cell transcriptomes in the intra-tumoral and associated territories of kidney cancer

**DOI:** 10.1016/j.ccell.2022.11.001

**Published:** 2022-12-12

**Authors:** Ruoyan Li, John R. Ferdinand, Kevin W. Loudon, Georgina S. Bowyer, Sean Laidlaw, Francesc Muyas, Lira Mamanova, Joana B. Neves, Liam Bolt, Eirini S. Fasouli, Andrew R.J. Lawson, Matthew D. Young, Yvette Hooks, Thomas R.W. Oliver, Timothy M. Butler, James N. Armitage, Tev Aho, Antony C.P. Riddick, Vincent Gnanapragasam, Sarah J. Welsh, Kerstin B. Meyer, Anne Y. Warren, Maxine G.B. Tran, Grant D. Stewart, Isidro Cortés-Ciriano, Sam Behjati, Menna R. Clatworthy, Peter J. Campbell, Sarah A. Teichmann, Thomas J. Mitchell

**Affiliations:** 1 Wellcome Trust Sanger Institute, Wellcome Genome Campus, Hinxton, Cambridge CB10 1SA, UK; 2 Molecular Immunity Unit, Department of Medicine, University of Cambridge, Cambridge CB2 0QQ, UK; 3 Cambridge University Hospitals NHS Foundation Trust and NIHR Cambridge Biomedical Research Centre, Cambridge CB2 0QQ, UK; 4 European Molecular Biology Laboratory, European Bioinformatics Institute, Wellcome Genome Campus, Hinxton, Cambridge CB10 1SA, UK; 5 UCL Division of Surgery and Interventional Science, Royal Free Hospital, London NW3 2PS, UK; 6 Specialist Centre for Kidney Cancer, Royal Free Hospital, London NW3 2PS, UK; 7 Department of Surgery, University of Cambridge, Cambridge CB2 0QQ, UK; 8 Department of Physics, Cavendish Laboratory, JJ Thomson Avenue, Cambridge CB3 0HE, UK

**Keywords:** renal cell carcinoma, kidney cancer, tumor microenvironment, single-cell sequencing, multi-regional sequencing, pseudocapsule, *IL1B*

## Abstract

Tumor behavior is intricately dependent on the oncogenic properties of cancer cells and their multi-cellular interactions. To understand these dependencies within the wider microenvironment, we studied over 270,000 single-cell transcriptomes and 100 microdissected whole exomes from 12 patients with kidney tumors, prior to validation using spatial transcriptomics. Tissues were sampled from multiple regions of the tumor core, the tumor-normal interface, normal surrounding tissues, and peripheral blood. We find that the tissue-type location of CD8^+^ T cell clonotypes largely defines their exhaustion state with intra-tumoral spatial heterogeneity that is not well explained by somatic heterogeneity. *De novo* mutation calling from single-cell RNA-sequencing data allows us to broadly infer the clonality of stromal cells and lineage-trace myeloid cell development. We report six conserved meta-programs that distinguish tumor cell function, and find an epithelial-mesenchymal transition meta-program highly enriched at the tumor-normal interface that co-localizes with *IL1B*-expressing macrophages, offering a potential therapeutic target.

## Highlights

• Single-cell and spatial sequencing reveals key features of renal cell carcinoma (RCC)
• Intra-tumoral heterogeneity of CD8^+^ clonotypes dwarfs that from somatic mutations
• RCC cells showing epithelial-mesenchymal transition (EMT) enrich at tumor edges
• Macrophages expressing *IL1B* correlate with EMT and could have therapeutic importance

## Graphical abstract

**Figure:**
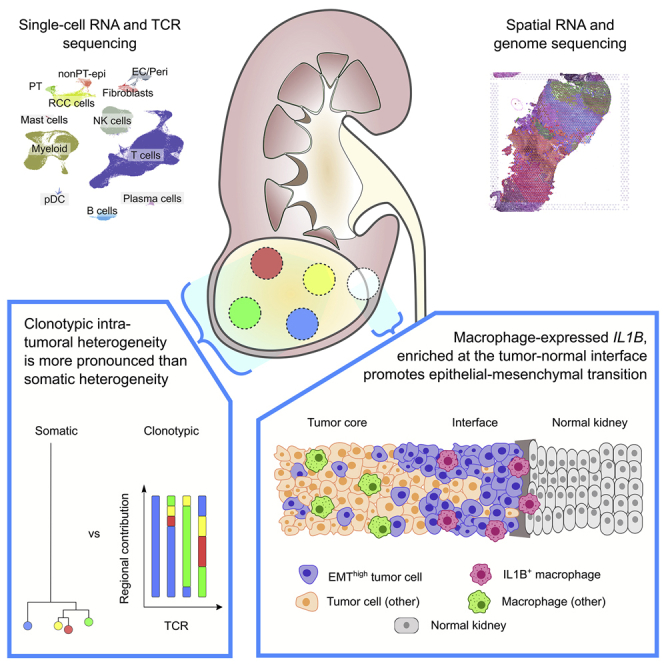

## Teaser

Li et al. use single-cell and spatial sequencing to examine the cellular features of kidney tumors and classify cancer cells according to function. They find a more invasive phenotype at the interface separating tumor with normal kidney, which appears to be driven by IL-1β signaling in macrophages.

## Introduction

Clear cell renal cell carcinoma (ccRCC) is the most common subtype of renal cell carcinoma (RCC), accounting for approximately 75% of RCC cases and the majority of deaths from kidney cancer.^1^ Many efforts have characterized the genomic landscape of ccRCC, revealing important driver events such as biallelic inactivation of *VHL*, followed by mutations in chromatin remodeling and histone modification related genes *PBRM1*, *BAP1*, and *SETD2*.^2^^,^^3^^,^^4^^,^^5^^,^^6^ Intra-tumoral heterogeneity (ITH) of these subsequent mutational events appears to be a salient feature of ccRCC, as revealed by previous multi-region exome sequencing studies.^5^^,^^7^^,^^8^ In contrast, the ITH of ccRCC at a transcriptional level is less well understood, in part due to the complexity of the multi-cellular ecosystem comprising the tumor microenvironment (TME). In particular, the phenotypic heterogeneity of malignant and non-malignant cells in the TME of ccRCC and how it associates with geographical localization remain elusive.

ccRCC is a cancer type with heavy infiltration of immune cells.^9^^,^^10^ Harnessing adaptive immunity through immune checkpoint blockade (ICB) therapy is effective in improving the survival of patients,^11^^,^^12^ highlighting the importance of the immune microenvironment of ccRCC. Characterizing this immune landscape using bulk sequencing is limited by the power to dissect diverse immune cell populations.^9^^,^^13^ A comprehensive single-cell immune atlas of ccRCC using mass cytometry shed light on immune cell diversity in the ccRCC tumor ecosystem.^14^ Recent advances in single-cell RNA sequencing (scRNA-seq) and its applications in cancer research have revolutionized our understanding of phenotypic heterogeneity of tumor cells,^15^^,^^16^^,^^17^ immune landscape of tumors,^18^^,^^19^^,^^20^ complexity and plasticity of the TME,^21^^,^^22^ and inter-cellular communications in the TME.^23^^,^^24^ Specifically, in ccRCC, a recent scRNA-seq study provided evidence to support its origin from proximal tubular cells.^25^ Other studies utilized scRNA-seq to study the immune landscape of ccRCC, mainly focusing on ICB-therapy-related cohorts^26^^,^^27^ and different disease stages,^28^ uncovering key features that are related to therapeutic efficacy or disease progression.

When considering heterogeneity of the TME, the geographic regions of interest extend beyond those relevant to mutational ITH. The wider regions of interest include circulating blood (as it is indicative of the systemic response to the local tumor and has implications for liquid sampling and inference of tumor behavior), the tumor-normal interface or tumor pseudocapsule (representing the boundary between tumor and adjacent normal kidney), adjacent normal kidney, and perinephric adipose tissue. The fibrous connective tissue comprising the pseudocapsule tends to constrain tumor growth spatially, and pseudocapsule invasion is correlated with tumor stage and grade.^29^ Perinephric adiposity is of interest because of the obesity paradox in RCC, whereby obesity is one of the strongest risk factors for the diagnosis of kidney cancer, yet is also associated with improved oncological outcomes.^30^ Understanding spatial heterogeneity and evolution of RCC with respect to tumor, immune and stromal cells, and their interactions in the wider TME is still lacking. To address this, we performed multi-region-based scRNA-seq from 12 patients, sampling peripheral blood, normal kidney, four different spatial regions of the tumor core, and the tumor-normal interface, alongside focally exhaustive exome sequencing of laser-capture microdissection (LCM)-derived tumor samples. We further validated important regional transcriptomic differences at finer resolution through the use of spatial transcriptomics, comparing cellular profiles across the tumor-normal interface with tumor core.

## Results

### Multi-region-based genomic and single-cell transcriptomic profiling of RCC

**Figure 1:**
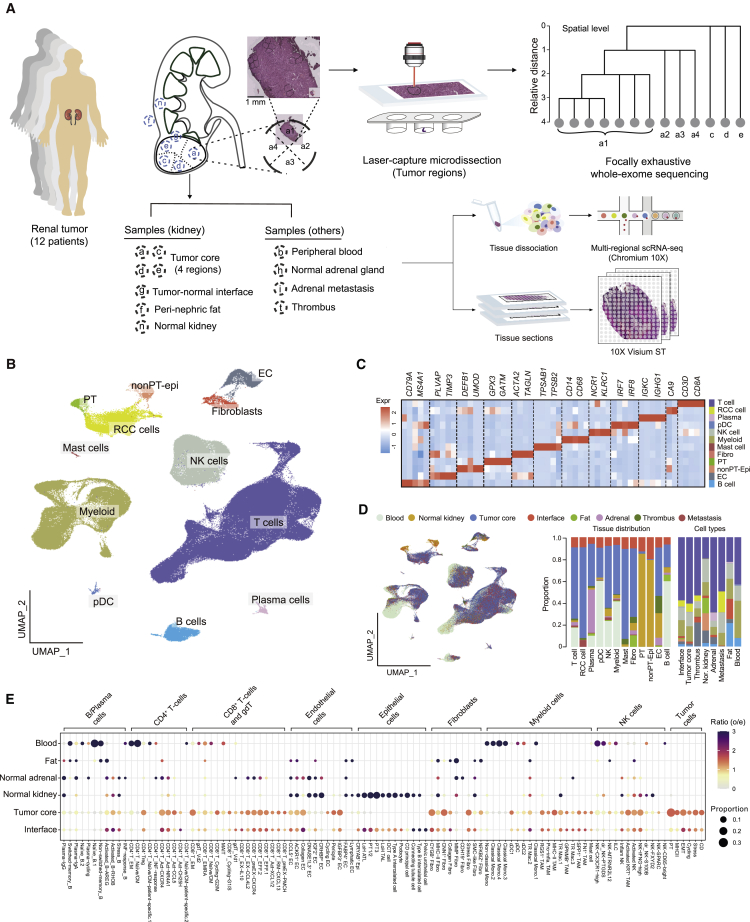
Sampling strategy and overall tissue distribution of the major cell types in RCC (A) Sampling strategy for each of 12 patient donors. a, c, d, and e represent four different regions of the tumor core; g, tumor-normal interface; f, perinephric fat; n, normal kidney; b, peripheral blood; h, normal adrenal gland; i, adrenal metastasis; t, thrombus. a1, a2, a3, and a4 represent LCM biopsies in tumor region a; ST, spatial transcriptomics. (B) Overall UMAP of all cells in our study. (C) Heatmap showing top differentially expressed genes (DEGs) in each of the major cell types. (D) UMAP and bar plots showing tissue distribution of the major cell types. Colors in the bar plots correspond to those in the UMAP here and in (B). (E) Dot plot showing tissue distribution of the fine-grained annotated cell types. Proportions are calculated as dividing cell numbers by total cell numbers of a certain major cell compartment. See also Figures S1 and S2; Table S2. Information on scRNA-seq and Visium spatial transcriptomics, related to Figure 1, Table S3. Information on LCM samples and WES, related to Figure 1, Table S4. Top differentially expressed genes in the subclustering analysis of different cell lineages, related to Figure 1

We conducted multi-region genomic and single-cell transcriptomic profiling in 12 patients, who underwent surgical resection of radiologically diagnosed and treatment-naive renal tumors, with the aim of sampling multiple low-, intermediate-, and high-risk tumors. After histopathological examination, tumors from 10 out of the 12 patients were evaluated as ccRCC, one (PD47172) was an oncocytoma, and one (PD44714) was a large benign thick-walled cyst (Figure S1A and Table S1). In each patient, we sampled tissues from peripheral blood, normal kidney, four geographically distinct regions of the tumor core, and the tumor-normal interface. Additionally we sampled tissues from the perinephric fat, normal adrenal gland, adrenal metastasis, and tumor thrombus, if available (Figure 1A). Where sufficient numbers of viable single cells could be retrieved, we performed droplet-based 5′ scRNA-seq with T cell receptor (TCR) enrichment using the 10X platform (Table S2). We also performed 10X Visium spatial transcriptomics on 11 tumor-normal interface and five tumor core tissue sections from eight patients (Table S2). In parallel, in each patient we dissected microbiopsy samples from each region containing tumor tissue using LCM prior to performing whole-exome sequencing (WES) (Table S3). Based on WES data, we identified genomic alterations that have been reported as recurrent/driver events in ccRCC.^2^^,^^4^ Seven out of nine ccRCC patients (no data in one ccRCC patient) harbored *VHL* mutations, four had *PBRM1* mutations, and three carried *BAP1* mutations (Figure S1A and Table S1). Copy-number loss of chromosome 3p was detected in all nine patients (Figure S1A).

**Figure 2:**
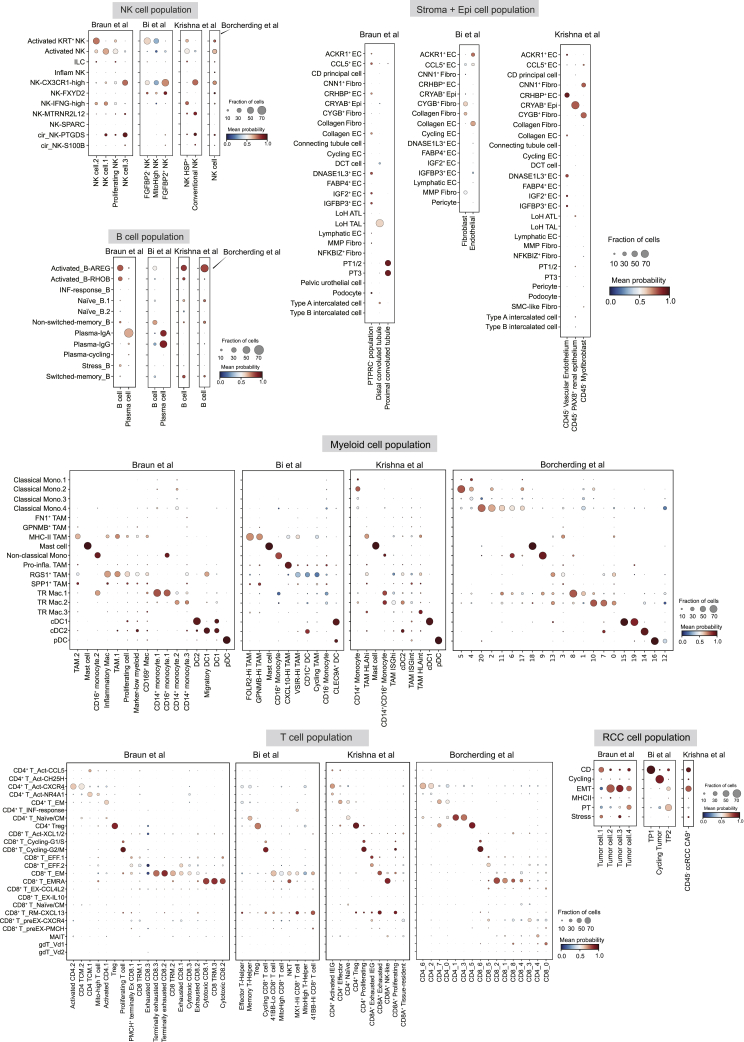
Cross-study comparisons of different cell types Cell types and annotations our study (rows) were compared with annotations or cluster numbers (columns) reported by four previous RCC studies, namely Biet al.,^26^ Krishna et al.,^27^ Braun et al.,^28^ and Borcherding et al.^31^ The comparison was based on logistic regression trained models using CellTypist^55^ with our data as the reference. Dot size represents the fraction of cells predicted as certain cell types, and color scale represents mean probability of prediction.

Using scRNA-seq, we captured transcriptomes from approximately 270,000 cells after stringent quality control, which can be broadly categorized into 12 major cell types based on the expression of canonical marker genes (Figures 1B, 1C, and S1B–S1D). As a result of our single-cell isolation protocol, T cells were most abundant in our data (Figures 1C and S1E). Tumor cells were identified within clusters that specifically expressed *CA9* and harbored extensive copy-number variations (CNVs) across their genomes, as inferred from scRNA-seq data (Figures 1C, S1E, and S1F). Next, we investigated the tissue of origin of the 12 major cell types and observed different tissue distributions (four tumor regions were combined in the analysis) (Figure 1D). We further conducted subclustering analyses for the major cell compartments, leading to the identification of 105 cell subsets with various tissue distribution preferences (Figure 1E and Table S4). Through a cross-study comparison covering four recently published scRNA-seq datasets,^26^^,^^27^^,^^28^^,^^31^ we showed that we substantially improved the characterization of the TME with refined cell-type annotations (i.e., for tumor cells) and newly reported cell types (i.e., gamma delta T [gdT] cells) (Figure 2).

Subclustering of natural killer (NK) cells revealed well-known *NCAM1-* and *FCGR3A-* expressing subsets and an interesting keratin (*KRT81* and *KRT86*)-expressing subset potentially enriched in tumor tissues (Figures 1E, S2A, and S2B; Table S4). In the B/plasma cell compartment, we identified major subsets including naive, switched memory, and non-switched memory B cells as well as plasma immunoglobulin A (IgA) and IgG cells (Figures S2C and S2D; Table S4). We observed heterogeneous stromal cell populations in our dataset (Figures S2E–S2H), which were largely under-reported in previous studies (Figure 2). In the endothelial cell (EC) compartment, we identified *IGFBP3*^+^ EC and collagen EC subsets showing considerable enrichments in tumor tissues (Figures 1E, S2E, and S2F). Collagen EC was more enriched in the interface, which may play roles in TME interactions through extra-cellular matrix (ECM) production. Similar to this, we also found a collagen-expressing fibroblast subset potentially enriched in the interface (Figures 1E and S2H). This suggests that different ECM-producing stromal cells tend to enrich and co-localize in the interface, possibly exerting diverse functions including extra-cellular context remodeling and cell-cell interactions. The normal epithelial cell population in our dataset exhibited expected diversity as reported previously^32^ (Figures S2I and S2J; Table S4).

### Expansion of CD8^+^ T cell clonotypes and the influence of tissue localization on exhaustion

**Figure 3:**
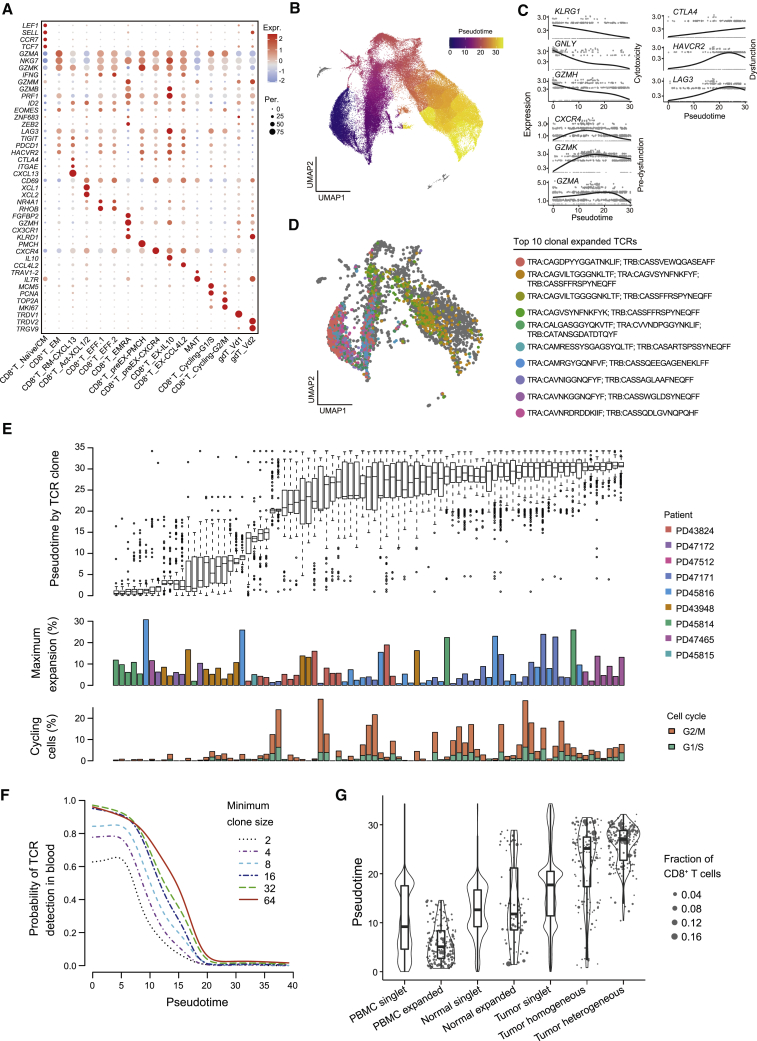
CD8^+^ T cell characterization, clonality, exhaustion, and regional enrichment (A) Dot plot showing marker gene expression defines principal CD8^+^ cell types. EM, effector memory; Act, activated; EFF, effector; EX, exhausted. (B) UMAP depicting the pseudotime inference of CD8^+^ cells. (C) Expression of canonical exhaustion markers across cells ordered by pseudotime analysis. All marker genes are statistically significant across pseudotime values (q value = 0; Moran’s I test in Monocle 3). (D) UMAP showing the ten most expanded clones from patient PD43948. Gray dots represent cells outside the ten most expanded clones. (E) Box plot depicting the most expanded clonotypes (>100 cells) across all patients, ordered by their mean pseudotime values, showing the median, interquartile range, and outlier pseudotime values. Statistical analysis by two-sided Wilcoxon rank-sum test. Data are presented as boxes with the median ± first and third quartiles with notches depicting 95% confidence intervals (top panel); bar plots showing the maximum expansion for the most expanded region (middle panel); and percentage of cycling cells (lower panel). (F) The probability of detecting a given TCR clone in peripheral blood as a function of minimal clone size and mean pseudotime value of the clone. (G) Mean pseudotime values based on the categorization of clonotypes according to their principal region of enrichment. Data are presented as median ± first and third quartiles with whiskers depicting 95% confidence intervals. Statistical analysis by Tukey’s test. See also Figure S3.

Subclustering of the CD8^+^ T cell compartment (including gdT cells), we identified typical CD8^+^ T cell clusters which represented different T cell functional states including naive, effector, memory, pre-dysfunction, and dysfunction based on the expression of canonical marker genes (Figures 3A, S3A, and S3B; Table S4). We identified resident memory T (TRM) cells highly expressing tissue-residency markers (i.e., *ITGAE* and *CD69*) and specifically expressing *CXCL13* among all cell types (Figures 3A and S3C). A similar CXCL13^+^ CD103 (*ITGAE*)^+^ CD8^+^ T cell subtype was previously suggested to play potential roles in mediating B cell recruitment and tertiary lymphoid structure formation in human cancer.^33^ We found cluster 6 highly expressed *FGFBP2* and *CX3CR1*, and was substantially enriched in peripheral blood (Figures 3A and 1E); therefore, this cluster may represent recently activated effector memory T cells (CD8^+^ T_EMRA). Two exhausted T cell clusters (clusters 7 and 8) were identified on the basis of elevated expression of genes including *LAG3*, *TIGIT*, *PDCD1*, *HAVCR2*, and *CTLA4* (Figure 3A). Interestingly, we found that cluster 8 had the highest expression of *LAG3* and specifically expressed the immunosuppressive cytokine *IL10* (Figure 3A). *IL10*-expressing CD8^+^ T cells in RCC were not found in the previous four RCC single-cell datasets (Figures 2 and S3D). These cells may represent CD8^+^ T cells with extremely high effector and dysfunction levels, which exert regulatory functions by producing interleukin-10 (IL-10). Besides the conventional CD8^+^ T cell clusters, we identified two gdT cell clusters, gdT_Vd1 (expressing *TRDV1*) and gdT_Vd2 (expressing *TRDV2*), which were not reported in the previous four RCC studies (Figures 2 and 3A). We also performed subclustering analysis of the CD4^+^ T cell population, revealing various subtypes such as CD4^+^ naive/central memory and CD4^+^ regulatory T cells and their different tissue distributions (Figures S3E–S3H and Table S4).

Next, we conducted a pseudotime trajectory analysis on CD8^+^ T cells excluding gdT cells and cycling clusters (Figures 3B and S3I). Along the pseudotime trajectory, we found that cytotoxicity-related genes (i.e., *KLRG1*, *GNLY*, and *GZMH*) were gradually downregulated while dysfunction-related genes (i.e., *CTLA4*, *HAVCR2*, and *LAG3*) were gradually upregulated (Figure 3C). Typical T cell pre-dysfunction-related genes (i.e., *CXCR4*, *GZMK*, and *GZMA*) were initially upregulated and then went downward along the pseudotime trajectory (Figure 3C). Therefore, this pseudotime trajectory recapitulated the progression of CD8^+^ T cells from a cytotoxic state via a pre-dysfunctional state to a dysfunctional state, alongside which the degree of exhaustion gradually escalated (Figure S3J). Furthermore, projection of the top ten expanded TCR clonotypes onto the trajectory led to an observation that individual TCR lineages were usually restricted to a similar phenotypic state rather than distributing across the entire trajectory (Figure 3D). Across all tumors, we found that 90% of clonotypes with 23 cells or greater were confined within a range of pseudotime values (p < 0.05, Wilcoxon test). Highly expanded TCR clones with over 100 cells per clone were observed in multiple patients, where remarkably up to 30% of CD8^+^ T cells can derive from a single clonotype (Figure 3E). In contrast, TCR clonotypes in CD4^+^ populations were less expanded compared with those in CD8^+^ populations (Figure S3H). Many of the most expanded CD8^+^ TCR clones had considerable proportions of cycling cells, with the exception being observed in the less-exhausted clonotypes (Figure 3E). This finding demonstrates that the proliferation in highly exhausted T cells in RCC has not been completely arrested, similar to previous findings in melanoma.^19^

We examined whether the TCR clonotypes detected in the blood reflected those detected in other regions. We found that the average degree of exhaustion (inferred pseudotime) and the probability of detecting CD8^+^ TCR clones in the peripheral blood were strongly anti-correlated regardless of the clonal size, to the extent that exhausted clonotypes are seldom detected in the blood (Figure 3F). This finding is unexpected and indicates that tissue-resident exhausted CD8^+^ T cell clones do not appear to recirculate in peripheral blood. To further illustrate the relationship between T cell exhaustion, clonal expansions, and their tissue distributions, we categorized CD8^+^ T cells according to whether they were singlets or expanded and their principal tissue locations (blood, normal tissues, or tumor). Expanded T cells in tumor were further subcategorized into those that appeared in all tumor regions and those that did not (tumor homogeneous and heterogeneous). Notably, the phenotypic state of CD8^+^ T cells, in terms of the degree of exhaustion, showed a strong dependence on clonal expansion and tissue location (Figure 3G; all p < 0.05, Tukey’s test). Meanwhile, clones private to one tumor region were not significantly more exhausted than those shared between different regions (Figure 3G; p > 0.05, Tukey’s test).

### Spatial localization rather than intra-tumoral somatic heterogeneity primarily influences CD8^+^ clonotypic heterogeneity

**Figure 4:**
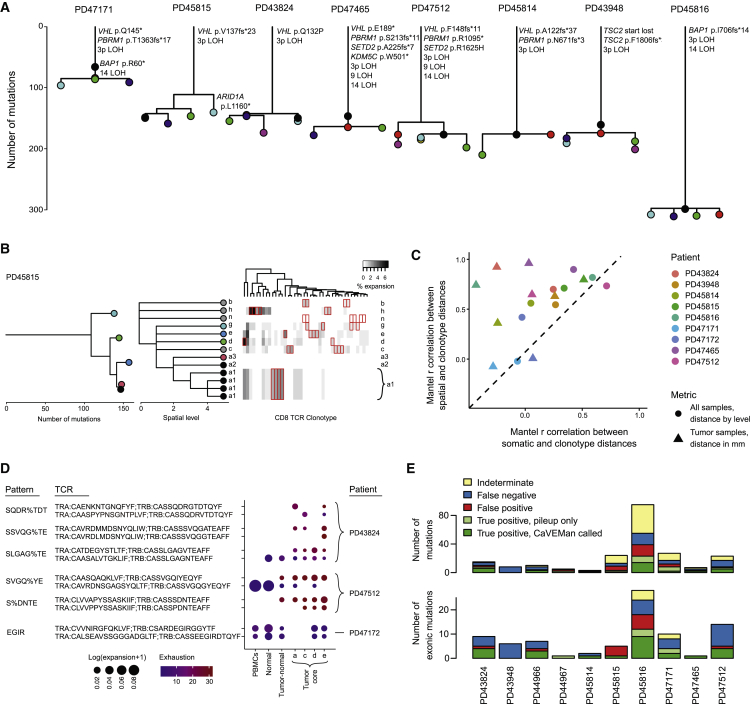
Somatic mutation calling and the relationship with TCR clonotypic heterogeneity (A) Reconstructed phylogenies from WES of multi-regional LCM biopsies. Each node represents a mutant clone present in one or more of the biopsies. (B) Comparison of WES-derived phylogenies (left) with geographic location (center) and CD8^+^ TCR clonotype expansion (right). Colors reference somatic clones to spatial localization. Each column in the right panel represents a TCR clonotype; those with significant regional enrichment are highlighted in red. a, c, d, and e represent four different regions of the tumor core; g, tumor-normal interface; n, normal kidney; b, peripheral blood; h, normal adrenal gland. (C) Scatterplot of Mantel correlation between tree distances. x axis represents the correlation coefficient between WES-derived clones and TCR clonotype distances. y axis represents the correlation coefficient between spatial localization and TCR clonotype distances. (D) Dot plot showing inferred TCR groups with a high probability of sharing antigen specificity. The exhaustion levels represent pseudotime values. (E) Benchmarking results for scRNA-seq-derived calls against WES data for each patient donor. See also Figure S4.

Using somatic mutations called from WES data, we constructed phylogenetic trees to elucidate the clonal evolution and ITH of the tumors in our study. Overall, we found that all tumor clones shared a long trunk but had short branches (Figure 4A). The majority of detected driver mutations and key CNVs were shared by all tumor clones within individual tumors (Figure 4A). Furthermore, the vast majority of LCM samples we sequenced appeared clonal according to the variant allele frequency distributions (Figure S4A). Taken together, the WES revealed that the extent of ITH of tumors in our cohort was limited. Previous studies have extensively investigated intra-tumor genetic heterogeneity in various cancers.^8^^,^^34^ However, the influence of somatic heterogeneity on the local TME at different spatial localizations, especially the anti-tumor immune response, remains largely uncharacterized. Here we investigated TME composition of the four tumor core regions which have similar histological grades and observed heterogeneity in cell compartments such as stromal cells (Figure S4B). This implies that the TME of RCC is spatially heterogeneous, orchestrated by various cell types, which may play roles in shaping tumor behaviors. On top of this, we systematically compared the relationship between somatic mutations, spatial localizations, and TCR clonotypes of CD8^+^ T cells in individual tumors (Figures 4B and S4C). We found that T cell clonotypes were often enriched in different tissues. Unexpectedly, tumor-associated clonotypes were frequently enriched in single regions, which appeared to harbor negligible heterogeneity of somatic mutations (Figures 4B and S4C). Somatic mutations, which generate neoantigens on tumor cells, are considered a driving factor for T cell clonal expansion upon antigen presentation. Our finding suggests that the heterogeneity of TCR clonal expansions associate more with the different spatial localization of T cells in tissues rather than ITH of somatic mutations. To formally examine this, we calculated the correlation between T cell clonotype distance and (1) mutation distance and (2) spatial localization distance (Figure 4C). We found that TCR heterogeneity in CD8^+^ T cells was more strongly correlated with spatial localization rather than somatic heterogeneity (p < 0.05, paired Wilcoxon test).

We sought to further understand the processes that are driving the tissue- and region-specific enrichment of many of the clonotypes. We used the GLYPH2 algorithm to cluster TCRs that are predicted to recognize the same epitope.^35^ In total, we detected six patterns that were shared between expanded clonotypes (Figure 4D). One of these patterns (SQDR%TDT) was enriched in different tumor regions for the two clonotypes. Two of the patterns (SLGAG%TE and SVGQ%YE) represented clonotypes that appeared at different stages at maturation, each with one clonotype that was present in peripheral blood and normal tissues and one that was enriched in tumor with high exhaustion levels. Taken together, these findings lend credence to the notion that there is ongoing priming of T cells, in part through the re-presentation of the same epitopes. The final region of residence within the tumor for these expanded clonotypes appears stochastically determined, perhaps influenced by local environmental factors at the time of seeding, but not uniformly distributed according to expression of the originally stimulating epitope.

### Precise *de novo* somatic mutation calling from scRNA-seq data

The detection of somatic mutations within single cells from their transcriptomic sequences may help infer their clonal relationships. We developed a framework to perform *de novo* somatic mutation calling from scRNA-seq data (deSCeRNAMut; see STAR Methods for more details) (Figure S4D). To benchmark our mutation-calling method, we first compared somatic mutations called from scRNA-seq data of tumor cells with those called from tumor WES data. Overall, our method achieved a good performance with a precision of 0.64 (or 0.70 when considering exonic mutations only) and a sensitivity of 0.53 (Figure 4E). We were also able to benchmark the method in CD8^+^ T cells, showing that 84% of called mutations are restricted to a single TCR clone (Figure S4E). This confirms the expected finding that the majority of mutations called in CD8^+^ T cells are restricted to clonotype because of the very limited number of mutations that could be shared between T cell clones prior to thymic maturation.

Using these mutation calls, we investigated the numbers of mutations expressed by different cell types, which can potentially shed light on their degree of clonal expansion. We calculated the proportion of cells with one, two, three, or greater than three mutations. We required at least 100 cells from each cell lineage and patient to account for the lack of discriminatory power in rarer cell populations (Figure S4F). As expected, the lineage with the highest number of cells expressing called mutations was the tumor cells, mainly explained by the known clonal structure of the lineage, but also due to the likelihood of increased mutational burden when compared with the normal cell types. For similar reasons, stromal cells did not typically have discernible numbers of cells with more than one called mutation. However, we observed a large number of myeloid cells expressing mutations, indicating that a sizable proportion of these cells are clonally related. This is perhaps unsurprising given the increasing incidence of clonal hematopoiesis with age^36^ and with a diagnosis of RCC.^37^ The majority of the mutations detected are likely to have been acquired during their hematopoietic stem cell state.^38^ The next cell types expressing mutations were fibroblasts, then CD8^+^ T cells (which we know are clonally expanded, based on the TCR-sequencing results). A very small proportion of CD4^+^ T cells expressed mutations, consistent with the low degree of clonality based on TCR analysis (Figure S3H).

### Regional characterization and evolution of myeloid populations

**Figure 5:**
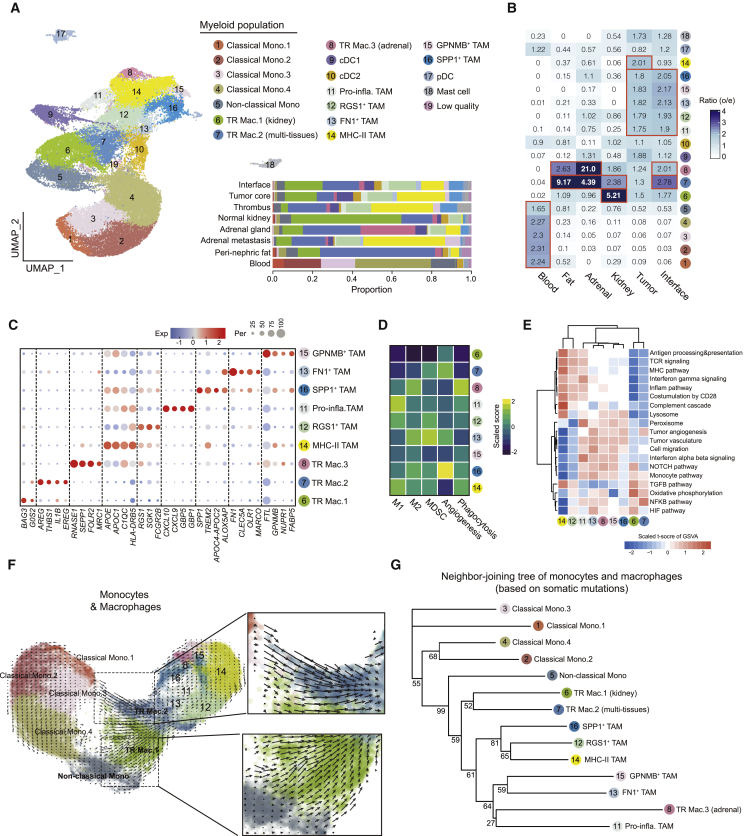
Myeloid cell characterization, regional enrichment, and evolution (A) UMAP re-presentation of all myeloid cells, their annotation, and their regional contribution. Mono, monocyte; TR Mac, tissue-resident macrophage; TAM, tumor-associated macrophage. (B) The relative enrichment of different myeloid cell subsets across different regions sampled. (C) Dot plot depicting top DEGs for macrophage clusters. (D) Heatmap showing mean scaled scores for macrophage subsets by macrophage function of M1/M2 polarization and suppressive, angiogenesis, and phagocytosis activity. (E) Heatmap showing the results of pathway enrichments of macrophage subsets using gene set variation analysis. (F) UMAP with superimposed RNA velocity analysis of the monocyte and macrophage subsets, with zoomed-in windows highlighting possible directional flows from monocytes to macrophages. (G) Neighbor-joining tree depicting the relationship of different monocyte and macrophage clusters, utilizing the somatic mutations called from scRNA-seq data. The numbers of supporting votes in bootstrapping (100 times) are labeled. See also Figure S5.

We captured heterogeneous myeloid subsets in subclustering analysis (Figures 5A and S5A; Table S4). Clusters 1, 2, 3, and 4 were predominantly present in the blood with high expression of *CD14* but lack of *FCGR3A* expression, thus representing circulating classical monocytes. Cluster 5 represented circulating non-classical monocytes with high expression of *FCGR3A* but lack of *CD14* expression (Figure S5B). We identified three dendritic cell (DC) clusters: plasmacytoid DC (pDC) and types 1 and 2 conventional DC (cDC1 and cDC2), characterized by specific expression of *JCHAIN*, *CLEC9A*, and *CD1C*, respectively (Figure S5B). We found mast cells, which were characterized by specific expression of *TPSAB1*, potentially enriched in the tumor core (Figures 5B and S5B), consistent with previous reports.^39^ Notably, we identified nine macrophage clusters (clusters 6–8 and 11–16) based on the high expression of *CD163* and *C1QC* (Figure S5B).

To further characterize the macrophage population, we explored differentially expressed genes (DEGs) and the tissue enrichment of the nine macrophage clusters, and compared the data with those from four previous studies (Figure S5C). We found six macrophage clusters (clusters 11–16) preferentially enriched in the tumor core/interface compared with other normal tissues, thus being defined as tumor-associated macrophages (TAMs). The remaining three clusters (clusters 6, 7, and 8) showed enrichment in normal tissues/interface and were regarded as tissue-resident macrophages (TR Mac) (Figure 5B). This distribution pattern can be also observed in the spatial transcriptomic data (Figure S5D). Among the six TAM clusters, MHC-II TAM (cluster 14) highly expressed *HLA-DRB5*, *APOE*, and *APOC1*, and was more enriched in tumor core than in tumor-normal interface. In contrast, the other five TAM clusters showed comparable degrees of enrichment in both tumor core and the interface (Figure 5B). *FN1*^+^ TAM highly expressed fibronectin 1 (*FN1*) and scavenger receptor *MARCO* (Figure 5C), which has been previously reported as a specific macrophage subset in kidney cancer.^39^ We found that *FN1*^+^ TAM was likely pro-tumor in RCC, as reflected by the high expression of a myeloid-derived suppressor cell signature and of M2 polarization genes (Figures 5D and S5E). We identified an *SPP1*^+^ TAM cluster expressing *GPNMB* that showed a high similarity to the *GPNMB*^+^ TAM identified by the previous study (Figure S5F). Considering we also identified a *GPNMB*^+^ TAM cluster (cluster 15) and that the expression of *GPNMB* can be detected in multiple TAM clusters (Figures 5C and S5G), this finding suggests that *SPP1*^+^ TAM may represent a subset of *GPNMB*^+^ TAM. Besides expressing *SPP1*, we found that *SPP1*^+^ TAM also expressed *APOC4-APOC2*, a gene not reported by previous RCC studies (Figure S5C), and *TREM2* (Figure 5C), which has been reported in various biological and pathological processes such as obesity and cancer.^40^^,^^41^

Among the three TR Mac clusters, TR Mac.2 was enriched at the interface (Figures 5B and S5D) and highly expressed interleukin *IL1B* and the epidermal growth factor receptor ligand *AREG*, which may reflect its likely role in tissue repair in homeostasis (Figure 5C). TR Mac.3 showed high expression of *SEPP1* and *MRC1*, and was extremely enriched in normal adrenal gland (Figures 5B and 5C). Interestingly, TR Mac.3 exhibited extremely high expression of M2 and phagocytotic signatures, and showed pathway activations similar to those of the pro-tumor TAM clusters (i.e., *FN1*^+^ TAM) (Figures 5D, 5E, and S5E). We were not able to clearly separate embryologically seeded from monocyte-derived tissue macrophages in this dataset.^42^

Using RNA velocity analysis, we found two obvious directional flows from circulating monocytes to macrophages in the tissue: (1) classical mono.3 to TR Mac.2 and (2) non-classical monocytes toward TR Mac.1 (Figure 5F). TR Mac.1 and TR Mac.2 then potentially gave rise to other macrophages in the tissues (Figure 5F). On the other hand, we leveraged the somatic mutations for lineage tracing, in a similar way to how the relationship of T cell phenotypic states has been determined from the sharing of TCR clonotypes. Here, by constructing a neighbor-joining tree (Figure 5G and STAR Methods), we found that circulating monocytes were separate from macrophages in tissues and that non-classical monocytes showed a closer relationship with macrophages in tissues when compared with other classical monocytes. Our data support non-classical monocytes representing an intermediary state between circulating monocytes and macrophages, with the majority of macrophages appearing to arise from monocyte progenitor rather than yolk sac origin.

### RCC expression meta-programs show differential abundance at the tumor-normal interface and correlate with prognosis

**Figure 6:**
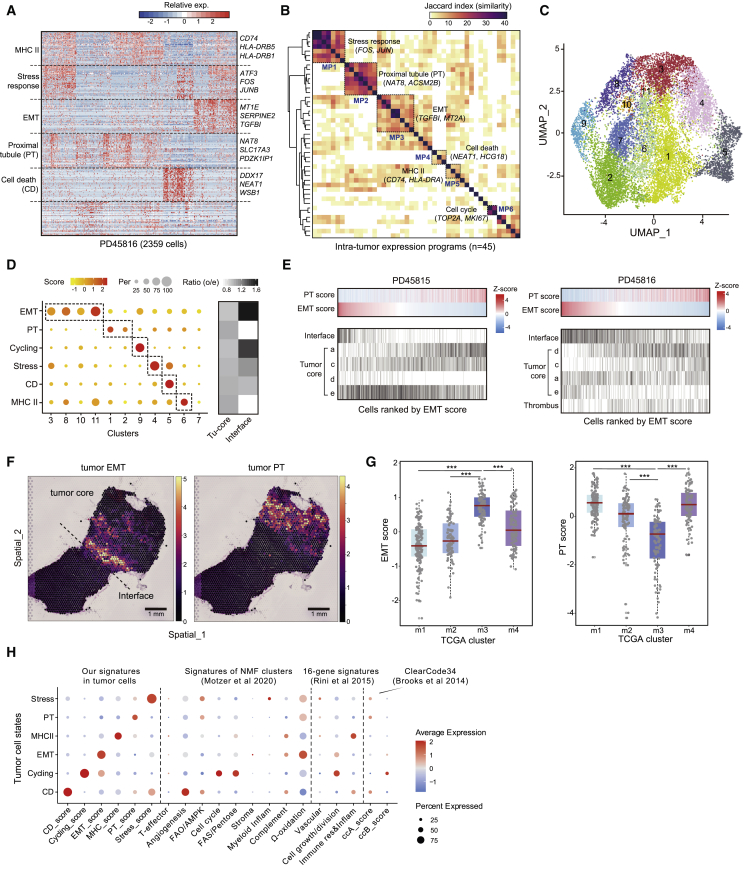
RCC cell expression programs, regional enrichment, and prognosis (A) Heatmap showing expression programs derived in a representative patient using NMF. (B) Heatmap depicting shared expression meta-programs across all patients. (C) UMAP representing clusters of tumor cell population. (D) Relative expression scores of meta-programs in each RCC cell cluster (left) and the distributions of cells with different meta-programs in tumor core and tumor-normal interface. (E) Cells from patient donors PD45815 and PD45816, ranked by decreasing EMT score with corresponding PT score and cell location. (F) Spatial mapping of EMT and PT tumor cells in a representative tumor-normal interface sample (PD47171) using cell2location. Estimated abundance (color intensity) is overlaid on a histology image. Scale bars, 1 mm. (G) Box plots showing the EMT and PT scores of TCGA samples in different molecular subtypes, which are m1 (n = 145), m2 (n = 90), m3 (n = 93), and m4 (n = 86). Data are presented as median ± first and third quartiles with whiskers depicting 95% confidence intervals. ^∗∗∗^p < 0.001 (two-sided Wilcoxon rank-sum test). (H) Dot plot showing gene scores of previously defined signatures in tumor cell populations. See also Figure S6 and Table S5.

To explore intra-tumor expression heterogeneity in the tumor cell population, we first defined intra-tumor expression programs that consist of co-expressed genes in each tumor using non-negative matrix factorization (NMF). These expression programs represented gene modules that were highly expressed by only subsets of tumor cells in each tumor, as exemplified by the NMF result in a representative tumor, PD45816 (Figure 6A). In total, we dissected 45 intra-tumor expression programs from the ten ccRCC tumors and classified six meta-programs (MPs) shared by multiple tumors (Figure 6B and Table S5). MP1 was characterized by expression of genes such as *FOS* and *JUN*, thus representing a stress-response-related signature in tumor cells. MP2 consisted of genes (i.e., *NAT8* and *ACSM2B*) that were specifically expressed by proximal tubule (PT) cells. The presence of PT signature among tumor cells confirmed the previous finding that PT cells are the cell type of origin of ccRCC.^25^ Interestingly, we found that MP3 was enriched for genes such as *TGFBI* and *MT2A* (Figure 6B), which are related to the epithelial-to-mesenchymal transition (EMT) and have not been reported in RCC previously (Figure 2). MP4 consisted of non-coding RNA genes such as *NEAT1* and *HCG18*, probably reflecting some stress or cell death (CD)-related cell state. MP5 was characterized by expression of MHC-II-related genes such as *CD74* and *HLA-DRA*. Genes such as *TOP2A* and *MKI67* were found in MP6, indicating that this MP is related to the proliferation of tumor cells.

Next, we integrated tumor cells from the ten tumors, mitigating the inter-patient heterogeneity through batch effect removal (Figures 6C and S6A). Through subclustering and DEG analysis, we validated the presence of the six MPs among tumor cells (Figure S6B). We calculated gene scores of the six MPs and mapped them onto the uniform manifold approximation and projection (UMAP) of tumor cells (Figure S6C). Interestingly, we found that the expression of PT and EMT programs showed an inverted pattern (Figure S6C), which was further confirmed by the anti-correlation between PT and EMT scores calculated in The Cancer Genome Atlas (TCGA) bulk RNA-seq data (Figure S6D). Furthermore, we found that EMT^high^ tumor cells were more abundant at the tumor-normal interface (the leading edge of a tumor) compared with the tumor core (Figure 6D), which reflects the fact that the EMT state represents a more invasive and migratory state of tumor cells. The relationship between PT and EMT programs and the spatial location of cells was exemplified by individual tumors (Figures 6E and S6E). We also observed this spatial distribution pattern of PT/EMT programs in the spatial transcriptomic data, after mapping cell types using cell2location^43^ (Figure 6F).

To investigate how our tumor cell signatures align with those previously defined and clinical related signatures, we first scored TCGA bulk RNA-seq data and found that the TCGA molecular subtype m3, which displays the worst prognosis according to TCGA study,^2^ showed significantly higher EMT scores but lower PT scores (Figure 6G). This finding indicates that cancer-specific survival may be linked to the relative abundance of these MPs within the tumor specimens (Figure S6F). We further checked the expression of three clinical related signatures in our tumor cells,^44^^,^^45^^,^^46^ and observed some extent of overlaps between signatures (Figure 6H). For example, CD^high^ tumor cells showed high expression of the angiogenesis signature defined by Motzer et al.^45^ and Cycling^high^ tumor cells showed high expression of the cell-cycle signature, the cell growth/division signature, and the FAS/pentose phosphate signature. EMT^high^ tumor cells have high expression of the Ω-oxidation signature and moderately high expression of the cell growth/division signature. We also realized that these clinically relevant signatures were mainly defined on the basis of bulk gene expression profile, thus some of them may reflect features of the TME. Therefore, we extended our analysis to all cell types/states identified in our study (Figure S6G), revealing some interesting findings. For example, the angiogenesis signature defined by Motzer et al.^45^ highlights six genes (*VEGFA*, *KDR*, *ESM1*, *CD34*, *PECAM1*, and *ANGPTL4*), but only four of them were expressed by endothelial cells while *VEGFA* and *ANGPTL4* were mainly expressed by tumor cells. We also found that *VEGFA* was also expressed in podocytes and *CD34* was expressed in matrix metalloproteinase (MMP) fibroblasts. These findings highlight that we can potentially refine these gene signatures by leveraging single-cell data and, in future use of these signatures, it might be useful to distinguish factors that are contributed by tumor cells or the TME.

### Interface enrichment and spatial correlation of *IL1B*-expressing macrophages with high EMT-expressing RCC cells

**Figure 7:**
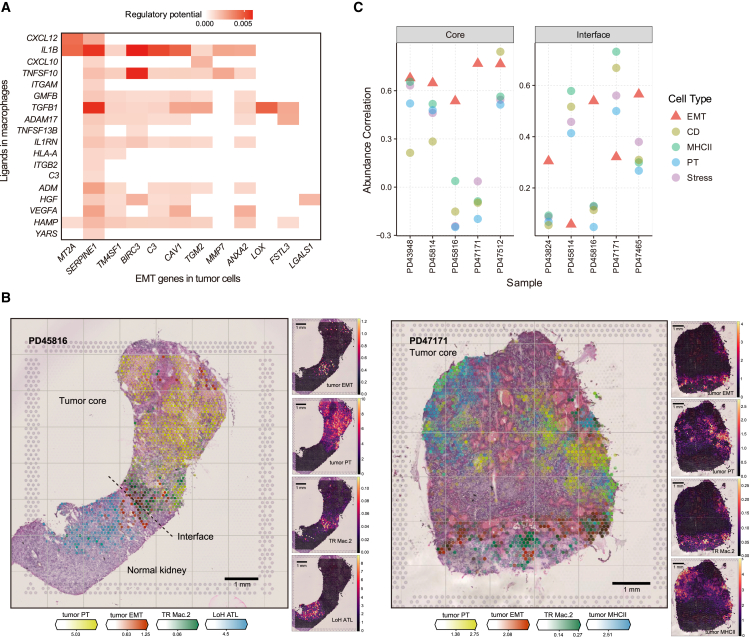
Cellular interactions in the ccRCC microenvironment (A) Heatmap depicting the potential regulation of genes expressed by the EMT meta-program and ligands expressed by macrophages. (B) Spatial mapping of EMT tumor cells, PT tumor cells, and TR Mac.2 in Visium data for representative tumor-normal interface (PD45816) and tumor core (PD47171) samples using cell2location. Estimated abundance for cell types (color intensity) across locations (dots) is overlaid on histology images. Scale bars, 1 mm. (C) Abundance correlation between EMT tumor cells and TR Mac.2 in tumor cores and tumor-normal interfaces from multiple tumors. See also Figure S7.

Our results indicated that EMT^high^ tumor cells preferentially localized to the leading edge of tumors (Figures 1E and 6D). This prompted us to explore whether there were any active inter-cellular interactions at the interface that could promote EMT in tumor cells. We used NicheNet^47^ to link ligands from cells in the TME and the EMT program in tumor cells. From this analysis, we found that macrophage-derived *IL1B* showed a high and wide regulatory potential to these EMT genes (Figure 7A), putatively via the receptor *IL1R1* expressed in tumor cells (Figures S7A and S7B). Interestingly, *IL1B* was specifically expressed by TR Mac.2 (Figure 5C), which again was enriched at the tumor-normal interface (Figure 5B).

We sought to validate this finding through spatial transcriptomics at the tumor-normal interface and tumor core. A consistent inverse correlation was again observed between signals derived from PT and EMT genes in RCC cells (Figures 6F, 7B, and S7C). Notably, we observed an enrichment of EMT^high^ RCC cells in close proximity to the tumor-normal interface. On further inspection, many of these regions co-locate with *IL1B*-expressing macrophages (TR Mac.2). We sought to formally quantify this correlation across all of our tissue sections by comparing the location of *IL1B* macrophages with all of the RCC cell subsets. We found that in many tissue sections, *IL1B*-expressing macrophages correlated most strongly with EMT^high^ RCC cells (Figures 7B and 7C). Surprisingly, this was true for all of the tumor core sections, which from our previous single-cell sequencing results showed a relative sparsity of EMT^high^ RCC cells. Among the tumor-normal interface sections, three out of five sections showed the strongest correlation between *IL1B*-expressing macrophages and EMT^high^ RCC cells.

Taken together, our findings indicate that *IL1B*-expressing macrophages (TR Mac.2) are co-localized with EMT^high^ RCC cells macroscopically and microscopically both at the tumor-normal interface and in the tumor core (Figures S7C and S7D). The microscopic correlation is not universal at the tumor-normal interface and may be a consequence both of the complex spatially dependent microenvironment and the challenges of accurate deconvolution of cell types from current spatial transcriptomic data. The ability of IL-1β expression to mediate RCC cell invasion has been previously investigated via a von Hippel-Lindau (VHL) null cell line model.^48^ Here, invasion of a collagen-rich matrix was induced by tumor cells via the IL-1β/CEBPβ/MMP pathway. In our data we also note that the EMT MP is defined by both *CEBPB* and *MMP7* (Figure S6B and Table S5). Our data support the notion that the IL-1β-mediated EMT pathway is promoting tumor growth, in part through facilitating the breakdown of the collagenase-rich interface.

## Discussion

We used multi-region-based genomic and single-cell transcriptomic sequencing to characterize the phenotypic heterogeneity and the multi-cellular ecosystem of ccRCC. Overall, our study depicts a comprehensive atlas of the TME of ccRCC alongside the established ITH in ccRCC, including the phenotypic categorization of tumor cells and immune/stromal cells and their inter-cellular communications in the TME, largely associating with their geographical localization.

Cells within expanded CD8^+^ TCR clonotypes were largely restricted by exhaustion levels. Similar observations were recently reported in melanoma.^49^ The phenotypic restriction of clonotypes may be related to either temporal maturation of clones or differential neo-epitope specificity rather than environmental factors, as individual tumors harbored clonotypes across the full diversity of states. We also found a spatial restriction of TCR clonal expansion within one or more of the macroscopic tumor biopsies. This cannot be entirely accounted for through the exposure to different mutation-associated neoantigens because of the limited observed ITH of somatic mutations, and that in some instances the same epitope binding patterns were enriched in different tumor regions (Figure 4D).

The utility of peripheral TCRs for non-invasive cancer detection and surveillance shows promise,^50^ especially in RCC where circulating tumor DNA fragments are scarce.^51^ Although we found many expanded clonotypes were represented both in blood and tumor regions, we observed that the degree of exhaustion and the probability of detecting TCR clones in the peripheral blood were inversely correlated to the extent that exhausted clonotypes are seldom detected in the blood (Figure 3F). This finding suggests that once T cell clones infiltrate into tumors and undergo phenotypic transition from activation to dysfunction they seldom recirculate, possibly due to a tissue-residency phenotype as evidenced by *CD69* (Figure 3A). Peripheral sampling of tumor-reactive TCRs is therefore more likely to either detect antecedents of exhausted tumor-resident clones rather than those currently active in the tumor, or to detect non-tumor-specific bystander clonal expansions. The tumor region and tissue-specific expansion of clonotypes has significant implications for the use of TCR sequencing in the detection and monitoring of disease. Our data also indicate that sampling of a single tumor region or tissue is unlikely to fully reflect the TCR clonal expansion in the entire tumor.

We developed a framework to accurately detect somatic mutations in different cell populations based on droplet-based scRNA-seq data. The principal challenges of the lack of consistent coverage, low read depth, and error-prone sequencing reads were abrogated using a number of filtering metrics including the implausibility of shared post-embryonic mutations between different cell-type lineages. We envisage that in the future, the use of spatial imaging techniques to visualize called mutations in expressed genes across a range of cell types will help to decipher the phylogenetic organization of the multi-cellular TME.

An EMT MP was defined and shared by multiple ccRCC tumors in our study. More abundant tumor cell populations and the use of methods to help circumvent challenging batch variations allowed us to uncover this previously unreported feature.^26^^,^^27^^,^^28^ EMT^high^ tumor cells in ccRCC tended to localize to the tumor-normal interface, which is the leading and migration edge of a tumor. These findings are similar to those reported in the scRNA-seq study of head and neck cancer.^16^

We identified that *IL1B*, specifically expressed by a subset of tissue-resident macrophage cells, could potentially promote tumor cells undergoing EMT, with both cell types found to be enriched at the tumor-normal interface. Expression of *IL1B* has been reported to positively correlate with tumor stages of RCC^52^ and is associated with worse prognosis of patients with RCC in patients recruited to TCGA. In addition, inhibition of IL-1β in RCC has been shown to induce tumor regression in a syngeneic murine model of RCC.^53^ IL-1β blockade has been shown to reduce incident lung cancer in patients with atherosclerosis,^54^ putatively preventing pre-existing clinically undetectable nascent tumor clones from progressing. Its use is now being investigated in several clinical trials, principally in later-stage disease. Our data indicate roles of macrophage-derived IL-1β signaling in RCC, acting through the promotion of EMT. Exploiting this pathway could be therapeutically useful, not only for those patients with established disease but also in the secondary prevention of cancer for those with RCC predisposition syndromes such as VHL disease.

## STAR★Methods

### Key resources table

**Table:** 

REAGENT or RESOURCE	SOURCE	IDENTIFIER
**Biological samples**		

Multi-regional tissue samples from renal cancer patients	This paper	Table S1. Clinicopathological information of patients in the cohort, related to Figure 1 and STAR Methods, Table S2. Information on scRNA-seq and Visium spatial transcriptomics, related to Figure 1, Table S3. Information on LCM samples and WES, related to Figure 1

**Chemicals**, **peptides**, **and recombinant proteins**		

PAXgene Tissue FIX Container	Qiagen	765312
PAXgene Tissue STABILIZER Concentrate	Qiagen	765512
Liberase TM	Roche	5401119001
DNase	Sigma	69182
RPMI	Gibco	21875034
gentleMACS	Miltenyi Biotec	130-093-237
70μm cells strainer	Falcon	10788201
Percoll	Sigma-Aldrich	P1644

**Critical commercial assays**		

Chromium single cell V(D)J enrichment kit, human T cell	10X Genomics	1000005
Chromium single cell 5′ feature barcode library kit	10X Genomics	1000080
Chromium single cell 5′ library and gel bead kit	10X Genomics	1000006
Chromium single cell chip A	10X Genomics	120236
Chromium I7 multiplex kit	10X Genomics	120262
Visium Spatial Tissue Optimization Slide & Reagents Kit	10X Genomics	1000193
Vsium Spatial Gene Expression Slide Kit	10X Genomics	1000184
PicoPure DNA Extraction Kit	Life Technologies	KIT0103

**Deposited data**		

Whole-exome sequencing raw data	This paper	EGAD00001008029
scRNA-seq raw data	This paper	EGAD00001008030
Spatial transcriptomics raw data	This paper	EGAD00001008781
scRNA-seq and spatial transcriptomics count data objects	This paper	Mendeley Data: 10.17632/g67bkbnhhg.1
TCGA data	Cancer Genome Atlas Research^2^	N/A
Myeloid data from Cheng et al	Cheng et al^39^	GSE154763
Data from Biet al	Biet al^26^	N/A
Data from Krishna et al	Krishna et al^27^	SRZ190804
Data from Braun et al	Braun et al^28^	N/A
Data from Borcherding et al	Borcherding et al^31^	GSE121638

**Software and algorithms**		

Samtools	Github	https://github.com/samtools/
Cell Ranger v2.1.1	10x Genomics	https://10xgenomics.com
Space Ranger v1.3.0	10x Genomics	https://10xgenomics.com
SoupX	Github	https://github.com/constantAmateur/SoupX
DoubletFinder	Github	https://github.com/chris-mcginnis-ucsf/DoubletFinder
Seurat v3.2	Stuart et al^56^	https://satijalab.org/seurat
InferCNV v1.6.0	Github	https://github.com/broadinstitute/inferCNV
Monocle 3	Github	https://github.com/cole-trapnell-lab/monocle3
CaVEMan v1.11.2	Github	https://github.com/cancerit/CaVEMan
Pindel v2.2.2	Github	github.com/cancerit/cgpPindel
AlleleCounter	Github	https://github.com/cancerit/alleleCount
ascatNGS	Github	https://github.com/VanLoo-lab/ascat
Dpclust	Github	https://github.com/Wedge-lab/dpclust
deSCeRNAMut	This paper	https://github.com/ThomasJamesMitchell/deSCeRNAMut
ANNOVAR	Wang et al^57^	https://annovar.openbioinformatics.org/en/latest
NicheNet v0.1.0	Github	https://github.com/saeyslab/nichenetr
scVelo v0.2.2	Github	https://github.com/theislab/scvelo
Scanpy v1.8.2	PyPI	https://pypi.org
Anndata v0.7.5	PyPI	https://pypi.org
numpy v1.19.5	PyPI	https://pypi.org
velocyto v0.17.17	Github	https://github.com/velocyto-team/velocyto.py
cell2location v0.7a0	Github	https://github.com/BayraktarLab/cell2location
CellTypist v1.1.0	Github	https://github.com/Teichlab/celltypist
NMF R package v0.23.0	CRAN	https://cran.r-project.org/web/packages/NMF
GSVA R package v1.38.2	Bioconductor	https://bioconductor.org/packages/release/bioc/html/GSVA.html
phangorn R package v2.5.5	CRAN	https://cran.r-project.org/web/packages/phangorn
ape R package v5.4	CRAN	https://cran.r-project.org/web/packages/ape
survival R package v3.2-3	CRAN	https://cran.r-project.org/web/packages/survival
survMisc R package v0.5.5	CRAN	https://cran.r-project.org/web/packages/survMisc
survminer R package v0.4.8	CRAN	https://cran.r-project.org/web/packages/survminer
GLYPH v2	Huang et al^35^	http://50.255.35.37:8080/

**Other**		

Web portal	This paper	https://www.sanger.ac.uk/project/microenvironment-of-kidney-cancer

### Resource availability

#### Lead contact

Further information and requests for resources and reagents should be directed to and will be fulfilled by the lead contact, Thomas J Mitchell (tjm@sanger.ac.uk).

#### Materials availability

This study did not generate new unique reagents.

### Experimental model and subject details

#### Human subjects

Human kidney and tumor tissues were collected through studies approved by UK NHS research ethics committees. Written informed consent was obtained from all donors. All adult kidneys samples, except PD44967 were collected from patients enrolled in the DIAMOND study; Evaluation of biomarkers in urological disease (NHS National Research Ethics Service ref. 03/018). Tumor PD44967 was collected from a patient enrolled in Characterisation of the immunological and biological markers of Renal cancer progression (NHS National Research Ethics Service ref. 16/WS/0039). Tissue samples were acquired as part of the DIAMOND study “Evaluation of biomarkers in urological disease” - NHS National Research Ethics Service reference 03/018, whose infrastructure is part-funded by the NIHR Cambridge Biomedical Research Centre (BRC-1215-20,014) and CRUK Cambridge Center Urological Malignancies program (Cancer Research UK Major Center Award C9685/A25117). Tissue and blood processing was carried out in the Clatworthy Lab, based in the University of Cambridge Molecular Immunity Unit in the MRC Laboratory of Molecular Biology. More relevant information is summarized in Table S1.

### Method details

#### Tissue sampling

Peripheral blood was sampled on the day of the surgery prior to removal of the kidney tumor and placed on ice. The surgical specimen was directly taken from the operating room to histopathology in order to minimise the warm ischaemia time. Biopsies were sampled by local pathologists to include (where available) multi-regional tumor biopsies from 4 macroscopically disparate regions, the tumour-normal interface, normal kidney (distant to the tumor and close to cortico-medullary border), perinephric adipose tissue, and adrenal gland. The biopsy locations from the bivalved kidney were annotated. Locations for the multi-regional core biopsies were determined by the following factors. First, the likelihood of harvesting viable tumor cells for single cell RNA sequencing and intact DNA for exome sequencing. Second, we aimed to sample from regions as geographically spatially separated as possible, without the risk of disrupting the clinical histopathological diagnosis. Tissue samples were divided and either placed on wet ice for immediate transfer for generation of single cell suspensions, underwent formalin-free fixation for 24 h in PAXgene Tissue FIX containers before being 20 transferred to PAXgene STABILIZER solution for storage at −20°C, or snap frozen prior to storage at −80°C.

#### Generation of single cell suspensions

The fresh tissue samples were coarsely dissected using a single edged razor blade prior to digestion for 30 min at 37°C with agitation in a digestion solution containing 25 μg/ml Liberase TM (Roche) and 50 μg/ml DNase (Sigma) in RPMI (Gibco). Following incubation samples were transferred to a C tube (Miltenyi Biotec) and processed on a gentle MACS (Miltenyi Biotec) on program spleen 4 and subsequently lung 2. The resulting suspension was passed through a 70μm cells strainer (Falcon), and washed with PBS. Percoll (Sigma-Aldrich) density separation was used both as a strategy to remove dead cells and cellular debris, and also to enrich stromal components of the TME, whilst still being permissive for a proportion of RCC cells themselves. We added the cell pellet to 44% Percoll in PBS (PBS) prior to centrifugation at 800G for 20 min. The supernatant was removed and the pellet suspended in PBS prior to centrifugation for 5 min at 800G. The concentration of enriched live cells was calculated after counting with a hemocytometer with trypan blue staining.

#### Cell loading and 10x library preparation

Cells were loaded according to standard protocol of the Chromium single cell 5′mRNA kit with TCR library enrichment in order to capture approximately 14,000 cells/chip position. All the following steps were performed according to the standard manufacturer protocol. Sequencing of libraries used either the Illumina HiSeq or NovaSeq systems.

#### Initial processing of scRNA-seq data

After the conversion of CRAMs files into FASTQs using samtools,^58^ we used the 10X software package cellranger (version 2.1.1 and vdj) and the GRCh38 reference genome for processing the 5′ sequencing data. We used SoupX^59^ to return an adjusted count matrix to account for ambient RNA contamination per channel using the adjustCounts() function. We then used DoubletFinder^60^ to estimate the probability of a given droplet containing RNA from more than one cell. Given that our cell loading aimed to recover 14,000 cells per lane, we assumed an 11% doublet formation rate.

#### scRNA-seq merge and QC

Seurat^56^ V3’s implementation of Reciprocal PCA (RPCA) was used to reduce the computational expense in merging the patient specific scRNA-seq data. Cells with greater than 30% mitochondrial content, or expression of fewer than 200 genes were excluded from further analysis. We used relatively permission thresholds to avoid removing renal epithelial cells that are known to have relatively high mitochondrial contents. We used standard clustering metrics and the expression of canonical marker genes to broadly classify cells into the principal cell subsets; T and NK cells, B and plasma cells, myeloid cells, endothelial cells, epithelial cells (non-cancerous), fibroblasts, and cancerous RCC cells. Cell clusters expressing implausible combinations of cell lineage specific marker genes were labeled doublets and were excluded from further analysis.

#### Cell type sub-clustering and annotation

We performed sub-clustering analysis of various cell compartments using the Seurat pipeline. Briefly, we first pulled out each cell compartment using the subset() function based on the broad classification of cells. We then used regularised negative binomial regression to normalise UMI counts using the SCTransform() function in Seurat, with the percentage of mitochondria genes being regressed out. Principal component analysis (PCA) was performed using the RunPCA() function based on highly variable features generated by using the VariableFeatures() function. For the PCA of T cell population, we excluded TCR encoding genes from the list of highly variable features so that to avoid clusters driven by the expression of different TCR genes. Batch correction was performed in each cell compartment using the RunHarmony() function implemented in the R package harmony, with the batch key (parameter ‘group.by.vars’) being set as patients and the assay (parameter ‘assay.use’) being set as ‘SCT’. Next, we performed nearest-neighbour graph construction, cluster determination and nonlinear dimensionality reduction using the FindNeighbors(), FindClusters() and RunUMAP() functions, respectively. The ‘reduction’ parameter in the FindNeighbors() and FindClusters() was set as ‘harmony’. DE-Gs of different clusters were extracted using the FindAllMarkers() function. Cell clusters expressing implausible combinations of cell lineage specific marker genes were labeled doublets and were excluded from the analysis. Cell type annotation was based on the expression of canonical markers and DE-Gs in various clusters. The annotation of cell cycle phases in the T cell population was based on the previously reported phase specific genes.^17^

#### Pseudotime inference, TCR analysis

Single cell count data and associated metadata of CD8^+^ T cells was analyzed using Monocle3 (https://github.com/cole-trapnell-lab/monocle3) after removal of cycling, gamma delta and MAIT cells. Pre-processing used the function preprocess_cds() with a dimensionality of 100, prior to alignment with ‘align_cds’ and batch correcting by individual sample. Dimension reduction used the function reduce_dimension(), prior to fitting the principal graph using ‘learn_graph’ and then ordering the cells using ‘order_cells’, all using the default parameters. To visualise the relationship of canonical marker genes of CD8^+^ T cell exhaustion we used the function plot_genes_in_pseudotime(). All such genes were found to be differentially expressed across the single cell trajectory using the function ‘graph_test’ at a *q* value of 0.

To demonstrate the differentiation properties of cells within clonotypes, we selected the most expanded clonotypes. For ease of interpretation we selected those clonotypes that contained at least 100 CD8^+^ T cells. The median, interquartile range, minimum, maximum values, and outlier values of pseudotime were plotted by clonotype, ordered by mean pseudotime values. The percentage maximum expansion was calculated from the region that contributed the maximum percentage of CD8^+^ T cells for each clonotype. The percentage of cells cycling in either the G1/S or G2/M phases were also calculated for each clonotype. We sought to quantify the degree of restriction of TCR clonotypes to a range of pseudotime values, by calculating the Wilcoxon test statistic for each clonally expanded CD8^+^ T cell clone (clones with more than one cell), compared to all of the other CD8^+^ T cells. To determine the likelihood of detecting expanded TCR clones in the blood as a function of pseudotime we computed the conditional density of detection of any cells with a given TCR in the blood, with pseudotime, for minimal clone sizes of 2, 4, 8, 16, 32, and 64 cells.

#### Laser capture microdissection, library preparation, and low-input DNA sequencing

Laser capture microdissection and low-input DNA sequencing followed the protocol previously reported.^61^ Briefly, PAXgene fixed samples were subsequently embedded in paraffin using standard histological tissue processing. 16μm sections were cut, mounted onto PEN-membrane slides, and stained with Gill’s haematoxylin and eosin. Using the LCM (Leica LMD7), tumor regions were selected in order to perform focally exhaustive tumor sampling. The dissected cells were collected into separate wells in a 96-well plate. Tissue lysis was performed using Arcturus PicoPure Kit (Applied Biosystems).

Libraries were constructed using enzymatic fragmentation as described previously and subsequently submitted for whole-exome sequencing on the Illumina HiSeq X platform. Short insert (500bp) genomic libraries were constructed, flowcells prepared and 150 base pair paired-end sequencing clusters generated on the Illumina HiSeq X platform without PCR amplification. The average sequence coverage was 84X and 92X for tumor and normal dissection samples, respectively (Table S3).

#### Mutation calling from whole-exome sequencing

DNA sequencing reads were aligned to the GRCh 37d5 reference genome using the Burrows-Wheeler transform (BWA-MEM).^62^ Single base somatic substitutions were called using an in-house version of CaVEMan v1.11.2 (Cancer Variants through Expectation Maximisation, https://github.com/cancerit/CaVEMan). CaVEMan compares sequencing reads from tumor and matched normal samples and uses a naive Bayesian model and expectation-maximisation approach to calculate the probability of a somatic variant at each base. Small insertions and deletions (indels) were called using an in-house version of Pindel (https://github.com/cancerit/cgpPindel). Post-processing filters required that the following criteria were met to call a somatic substitution:

1. At least a third of the reads calling the variant had a base quality of 25 or higher.
2. If coverage of the mutant allele was less than 8, at least one mutant allele was detected in the first ⅔ of the read.
3. Less than 5% of the mutant alleles with base quality ≥15 were found in the matched normal.
4. Bidirectional reads reporting the mutant allele.
5. Not all mutant alleles reported in the second half of the read.
6. Mean mapping quality of the mutant allele reads was ≥21.
7. Mutation does not fall in a simple repeat or centromeric region.
8. Position does not fall within a germline insertion or deletion.
9. Variant is not reported by ≥ 3 reads in more than one percent of samples in a panel of approximately 400 unmatched normal samples.
10. A minimum 2 reads in each direction reporting the mutant allele.
11. At least 10-fold coverage at the mutant allele locus.
12. Minimum variant allele fraction 5%.
13. No insertion or deletion called within a read length (150bp) of the putative substitution.
14. No soft-clipped reads reporting the mutant allele.
15. Median BWA alignment score of the reads reporting the mutant allele ≥140.

The following variants were flagged for additional inspection for potential artifacts, germline contamination or index-jumping event:

16. Any mutant allele reported within 150bp of another variant.
17. Mutant allele reported in >1% of the matched normal reads.
18. The median alignment score of reads that support a mutation should be greater than or equal to 140 (ASMD ≥140)
19. Fewer than half of the reads should be clipped (CLPM = 0).

We then tested for true presence or absence of the somatic variants that passed the above flags using an approach previously described.^63^ Briefly, counts were re-calculated using AlleleCounter (https://github.com/cancerit/alleleCount) across all the samples in this study. For each patient, the non-tumour samples in this study not belonging to that patient were used as a reference to obtain the locus-specific error rate. To minimise the false positive rate, the presence of the variant in the sample was accepted if the multiple-testing corrected p value was less than 0.001. The ascatNGS^64^ algorithm was used to estimate tumor purity and ploidy and to construct copy number profiles. A penalty of 200 was used with the prior knowledge that copy number events in RCC tended to be either arm or chromosome level.

#### DNA mutational clustering

Mutations were clustered using a Bayesian Dirichlet based algorithm as described previously.^65^ Briefly, the expected number of reads for a given mutation if present in one allelic copy of 100% of tumor cells may be estimated based upon the ASCAT derived tumor cell fraction, the copy number at that locus and the total read-depth. The fraction of cells carrying a given mutation is modeled by a Dirichlet process with an adjustment for the decreased sensitivity in identifying mutations in lower tumor fractions. Mutations were thus assigned to clusters according to the calculated fraction of clonality. The hierarchical ordering of these clusters was determined by applying the pigeonhole principle.

#### *De novo* Mutation calling from scRNA-seq data

The code for this method is available at https://github.com/ThomasJamesMitchell/deSCeRNAMut. The steps are described below:

##### Initial variant calling

In order to call cell specific mutations, indexed BAM files from the cellranger pipeline were first split into cell specific BAM files and were indexed using samtools.^58^ Mutations were initially called using bcftools mpileup. The choice of mutation caller was primarily influenced by the need for high sensitivity calls of variants with few supporting reads.^66^ Unsurprisingly, a huge number of mutations were called - with between 800,000 and 4,000,000 mutations called per patient. To facilitate more efficient downstream filtering of putative mutations, we perform the first filter step at this point:

• Removal of singlet variants only called in a single cell as it will be challenging to accurately determine whether these mutations are real or artifact.
• Removal of variants that are shared between the main cell lineages of T and NK cells, B and plasma cells, myeloid cells, endothelial cells, epithelial cells (non-cancerous), fibroblasts, and cancerous RCC cells. The vast majority of somatic mutations are acquired post embryonic differentiation, and therefore any true degree of sharing is implausible.

After these steps, we are left with between 40,000 and 300,000 mutations per patient. We have generated a list of putative variant sites, but we are unaware how many variants may have been missed at each loci, and we have no information regarding reference calls at those loci. We therefore run alleleCount (https://github.com/cancerit/alleleCount) to generate count tables of each base for all cells at every putative patient-specific loci.

##### Collation and annotation of counts

Reference and variant counts were collated for all of the loci called above to create a sparse matrix of counts for all cells. In the absence of copy number variants, if an autosomal chromosome harbors a true mutation, one expects an approximately equal number of reference and variant calls. The exception is for genes that exhibit a high degree of allelic specific expression, or that typically transcribe a particular allele in concentrated bursts. Alternatively, a high ratio of reference to variant counts in a cell base may imply artifact associated with high depth sequencing/poorly mapped regions. A binomial filter (p < 0.05) was therefore applied in each cell, with calls ignored in future analyses if there are significantly higher reference than variant counts.

Each genomic loci was annotated using ANNOVAR^57^ and the trinucleotide context of the variant.

The number of cells containing either the reference or variant base were collated for:

• The cell lineage with the greatest number of mutations.
• All of the other cell lineages.
• The TCR clonotype with the greatest number of mutations.
• All other TCR clonotypes

Fisher’s exact test was used to compute whether there are proportionally greater numbers of mutations in the cell lineage/clonotype with the greatest number of mutations. An enrichment factor was also calculated for each mutation that represents the multiple of the increased prevalence in the predominant cell type compared to all others.

##### Final filter

We applied the following thresholds to filter all possible mutations
• Fisher’s exact significance of enrichment by cell lineage, *p* < 0.0001 with proportionally at least 5 times greater mutations in the most enriched lineage.
• Absence of any known single nucleotide polymorphisms from either ExAC or dbSNP.
• No shared mutations between patients
• Adequate coverage with at least 5 cells with variant base from the mutated cell lineage and at least 20 cells with reference base from the reference population

We then examined the trinucleotide context of called mutations after this filtering step. Note is made of high levels of mutations that are otherwise unexplained from published catalogs of mutational signatures (particularly in a GCN > GGN and GTN > GGN context). By separating the trinucleotide context into the positive versus negative transcribed strands, we see differences that are otherwise unexplained by DNA derived mutational signatures, implying artifact either through library prep, sequencing, or RNA editing.

The striking strand bias cannot be accounted for by known mutational processes. Given the disparity between transcribed strands, mutations that have arisen with a highly biased context are removed (binomial filter, p < 0.005). We finally removed all mutations that are clustered within 4 bases in a given patient, to yield the final mutation calls.

##### Benchmarking data by whole-exome sequencing

Multi-regional whole exome sequencing data has been processed for tumor tissue adjacent to the regions that have undergone single cell RNA sequencing. The exonic mutations may therefore be used as a benchmark to determine the precision and sensitivity of the single cell mutation calling method above. To provide a fair comparison between single cell RNA and bulk exonic DNA mutation calls, and to account for differences in coverage between the methods, we also examine whether there is evidence of a given mutation using the reciprocal technology by performing a pileup at that mutation locus.

We can therefore classify mutations called using the above pipeline as:

• True positive - The mutation has been called in both in the scRNA-seq pipeline and CaVEMan.
• True positive, pileup only - The mutation has been called in the scRNA-seq pipeline, and there is evidence of the mutation in exome sequencing from tumor regions, with no mutations in the normal sample BAM files. The most common reasons for these mutations not being called by CaVEMan is low coverage or the mutation being called in mtDNA.
• False positive - The mutation has been called in the scRNA-seq pipeline, but there are fewer than 5 supporting reads for the variant base, and more than 20 reads for the reference base in the exome data.
• False negative - The mutation has been called by CaVEMan from the exome data, and has not been called from the scRNA-seq data, despite there being adequate coverage of at least 5 cells with the variant and at least 20 cells with the reference base.
• Indeterminate - The mutation has been called by the scRNA-seq pipeline, but there is not sufficient depth in the exome data to corroborate the call.

Note that it is possible that some of the false positive results may be real mutations that simply have not been captured spatially as adjacent tissue was sequenced. Overall, this scenario is unlikely as the majority of mutations are clonal and present throughout the tumor.

##### Benchmarking data by clonotype

In adult tumors, one expects a high proportion of somatic mutations in expanded CD8^+^ T cells to have been acquired post thymic selection. Most called mutations should therefore be restricted to a single T cell receptor clonotype. By using identical metrics to those used to select mutations across all cell types, we examined the proportion of CD8^+^ T cell mutations that are restricted to a single clonotype. Again, in order to call a mutation, we use thresholds requiring at least 5 cells with the variant in the most prevalent clonotype, with a least 20 cells covering the reference allele in the other clonotypes.

#### Inferring copy number variations based on scRNA-seq data

To effectively distinguish malignant and non-malignant cells, we inferred the large-scale chromosomal CNVs of single cells based on scRNA-seq data using the tool InferCNV (https://github.com/broadinstitute/inferCNV) with default parameters. Briefly, InferCNV first orders genes according to their genomic positions (first from chromosome 1 to X and then by gene start position) and then uses a previously described sliding-average strategy to normalise gene expression levels in genomic windows with a fixed length. Multiple putative non-malignant cells are chosen as the reference to further denoise the CNV result. In our analysis, we chose epithelial cells (including both PT and non-PT cells), endothelial cells and fibroblasts as the reference cell types to define a baseline in inferring CNVs.

#### Cell subtype abundance in different tissues

To explore the potential enrichments of cell subtypes in different tissues, we compared the observed and expected number of cells of all cell types/subtype across different tissues. Adrenal metastasis and tumor thrombus were excluded from this analysis as we only managed to sample them in single patients. The ratio of observation to expectation (R_O/E_) was calculated as follows:

$$R_{O/E}=Observed/Expected$$

where the expected number of cells were calculated based on the Chi-square test. In this analysis, we excluded cells from the adrenal metastasis and tumor thrombus because we only captured cells from these two tissues from single patients. A specific cluster was considered as being enriched in a specific tissue if Ro/e > 1. In the dot plot shown in Figure 1E, all proportions were calculated as dividing cell numbers by total cell numbers of a certain major cell compartment. We filtered out proportions smaller than 0.001 to display the result.

#### Cross-study comparison analysis using the CellTypist to train LR models

To perform cross-study comparisons, we trained logistic regression (LR) models with our dataset and cell annotations as the training data using the CellTypist,^55^ an automated cell type annotation tool for scRNA-seq datasets on the basis of logistic regression classifiers optimised by the stochastic gradient descent (SGD) algorithm. The training process was conducted for each major cell compartment separately, in which we first performed a fast feature selection based on the feature importance (the absolute regression coefficients) using SGD learning and then re-ran the classifier using the corresponding subset genes of the input data. We used LR models for different cell compartments to predict the identities of cells in four previously published datasets,^26^^,^^27^^,^^28^^,^^31^ and compared the predicted cell identities to the provided annotations/cluster numbers. Among the four previous studies, Braun et al,^28^ Biet al^26^ and Krishna et al^27^ captured all major cell populations in the TME of RCC, including stroma, immune, epithelia and RCC cells, while Borcherding et al^31^ only profiled the immune cell compartment. We included all these cell populations in the comparison analysis.

#### Correlation between spatial, somatic RCC evolution and TCR clonotype evolution

Tree structures relating to somatic ITH, spatial localisation of the tissue samples, and CD8^+^ clonotype enrichment for each region sampled were generated. The distance matrix relating regions to their somatic ITH was generated using pairwise distances from the mutational cluster output from the Bayesian Dirichlet based algorithm from the WES data for each of the (clonally) derived LCM samples. The spatial localisation distance matrix was calculated from the pairwise distances from tree structures determined either by:

1) The approximate absolute distance between LCM biopsies: This metric is not meaningful for normal tissue samples, particularly for peripheral blood and therefore the normal samples were excluded using this absolute distance metric.
2) A categorical distance: The first level equates to adjacent LCM biopsies, whose centers lie approximately 0.2mm apart. The second level for LCM biopsies taken from the same histologically mounted section, approximately 2mm distant. The third level relates to biopsies from small macroscopically separate biopsies, separated by approximately 6mm. The fourth level relates to macroscopic tumor biopsies taken approximately 30mm apart. The fifth level encompasses all of the adjacent normal tissue samples.

The Euclidian CD8^+^ T cell clonotype distance matrix was calculated using the relative expansions of the CD8^+^ clonotypes for each region sampled. Regions were removed where there was incomplete data – for instance if there were no viable cells in the single cell sequencing data. However, any regions where there was overlapping data, for instance multiple WES data from adjacent LCM cuts relating to a single region for single cell RNA sequencing were all included.

The pairwise correlation between the above distance matrices was computed using the Mantel test. A paired Wilcoxon test was used to determine whether somatic ITH or spatial localisation correlated with CD8^+^ clonotypic heterogeneity.

#### Gene set enrichment analysis and gene signature scoring in macrophage population

We performed gene set variation analysis among macrophage subsets using the GSVA R package. The gene sets we used were the C2 collection (curated gene sets) downloaded from the MsigDB database (https://www.gsea-msigdb.org/gsea/msigdb). The differences in activities pathways between clusters were calculated using the Limma R package. Significantly disturbed pathways were identified with Benjamini-Hochberg–corrected p value of <0.01. Some representative pathways that related to tumor progression, immune response and regulation were selected to make a heatmap. We investigated the phenotypes of different macrophage subsets by scoring them based on four previously reported gene signatures, including M1 and M2 polarisation,^18^ signature of myeloid-derived suppressor cells (MDSC),^67^ and signatures of angiogenesis and phagocytosis.^39^

#### RNA velocity analysis

We conducted RNA velocity analysis using velocyto.^68^ We first ran the command line ‘velocyto run10x’ to annotate spliced and unspliced reads using the cellranger output (the BAM file) as the input, generating loom files for each cellranger output. We then merged these loom files and pre-processed the velocity data using the scVelo python package.^69^ We projected the velocity information onto pre-generated UMAP and visualised the results using the function scvelo.pl.velocity_embedding_grid().

#### Similarity analysis of myeloid clusters

To compare the similarities of myeloid clusters to the previously published data,^39^ we trained a logistic regression model using elastic net regularisation as previously described.^25^ The previous kidney cancer data were obtained from Gene Expression Omnibus (GEO: GSE154763) and were used as training data.

#### Lineage tracing using scRNA-seq called somatic mutations

Based on the somatic mutations called from scRNA-seq data, we constructed a neighbour-joining tree to elucidate the relationship of different monocyte and macrophage subtypes (the low-quality cluster was excluded). Since our somatic mutations were called from gene expression data, we realised that the expression levels of genes may impact on the detection of mutations in different clusters, thus potentially making cell subtypes with more similar expression profiles cluster closer while those with less similar expression profiles segregate farther in the tree structure. To mitigate this, we excluded mutations that were detected in the top 100 DE-Gs of every cluster from the tree construction process. Based on the remaining mutations, we created a mutation matrix (mutation × subtype) considering whether a specific mutation appears in specific subtypes or not. Next, we calculated the binary distance between any two cell subtypes based on the mutation matrix and constructed the neighbour-joining tree using the ‘NJ’ function in the R package ‘phangorn’. A bootstrapping analysis was performed using the ‘boot.phylo’ function implemented in the R package ‘ape’, with the number of bootstrap replicates being set as 100. The final tree structure was displayed using the ‘plotBS’ function in the R package ‘phangorn’.

#### Deciphering intra-tumour expression programmes and meta-programmes

To explore underlying intra-tumour expression signatures of tumor cell population in RCC, we applied non-negative factorization (implemented in the R NMF package) to the tumor cells in ten patients (PD44714 and PD47172 were excluded from this analysis because they were histologically evaluated as benign and oncocytoma). Briefly, for each tumor, we first normalised the expression counts using Seurat NormalizeData() function with default parameter settings. We selected highly variable genes (HVGs) using Seurat FindVariableFeatures() function and only focused on the 2000 HVGs in downstream analysis. Then, we performed centre-scale for HVSs using Seurat ScaleData() function with the percentage of mitochondria genes being regressed out, and replaced all negative values in the expression matrix by zero. The top 10 ranked co-expressed gene modules in each tumor sample were dissected by using the nmf() function in the NMF package. For each gene module, we extracted the top 50 genes with the highest weight and used them to define a specific intra-tumour expression program. Finally, we only included those expression programmes with standard deviations larger than 0.2 among tumors cells, thus generating 3 to 6 intra-tumour expression programmes in the 10 tumors.

To investigate if some intra-tumour expression programmes were actually shared by multiple tumors, we applied a clustering analysis to all programmes based on the pair-wised Jaccard index calculated as follows, where A and B represent two intra-tumour programmes.

Jaccardindex=A∩B/A∪B

We defined those intra-tumour programmes shared by multiple tumors as meta-programmes. Genes that are shared by at least 50% tumors with a specific meta-programme were used to define the meta-programme except for the cell cycle program, which is only shared by two tumors and thus we used genes shared by the two tumors to define the cell cycle program.

#### Integrating and analysing tumor cells from different patients

To mitigate the effect brought by the strong inter-tumour heterogeneity in integration, we used the Seurat scRNA-seq integration pipeline to integrate tumor cells from 10 patients (PD44714 and PD47172 were excluded from this analysis because they were histologically evaluated as benign and oncocytoma). Briefly, for each tumor, we first used regularised negative binomial regression to normalise UMI counts based on the SCTransform() function in Seurat with the percentage of mitochondria genes being regressed out. The pre-processed individual objects were then added to a list, based on which we further performed selection of integration features using the SelectIntegrationFeatures() function with the number of features being set as 3000. We next performed integration preparation using the PrepSCTIntegration() function and found the integration anchors using the FindIntegrationAnchors() function with the normalisation method being set as ‘SCT’ and the ‘k.filter’ parameter being set as 50. Finally, these objects were integrated by using the IntegrateData() function. Based on this integrated object of tumor cells, we further performed downstream analyses including clustering and differentially expressed gene analysis. Gene signature scores of the six identified meta-programmes were calculated with the AddModuleScore() function using featured genes in these programmes.

#### TCGA data and prognosis analysis

We used TCGA expression and prognostic data to calculate meta-programme scores and investigate how the meta-programmes correlate with survival of patients with ccRCC. We processed the gene expression matrix by log-transforming and centralising. Gene scores of each meta-programme were calculated as the average expression of genes in the specific program. TCGA samples with records of age, gender, stage, survival data and tumor purity information were further used for survival analysis. For the expression of each meta-programme, patient cohorts were grouped into high and low groups by the optimal cut point determined using the cutp() function documented in the survMisc R package. We performed multivariate analyses using the Cox proportional hazards model (coxph() function in the survival R package) to correct clinical covariates including age, gender, tumor stage and tumor purity for all survival analyses in our study. Kaplan-Meier survival curves were plotted to show differences in survival time using the ggsurvplot() function in the survminer R package.

#### Cell-cell interaction analysis

To study if any active intercellular interactions at the interface that potentially promoted EMT in tumor cells, we conducted an analysis of cell-cell interaction by linking ligands expression on one cell type to some target genes of interest expressing another cell type using NicheNet.^47^ This analysis uses public databases (KEGG, ENCODE, PhoshoSite) to track downstream effectors such as transcription factors and receptor’s target in the provided dataset. Specifically, we were interested in what ligands from non-malignant cells in the TME can potentially trigger EMT program in tumor cells, thus considering the gene list of deciphered EMT meta-programme as the target genes. Genes were considered as expressed when they have non-zero values in at least 5% of the cells in a specific cell type.

#### Sample preparation for 10x Genomics Visium spatial transcriptomics

Fresh frozen samples from tumor core and tumour-normal interface tissues were first embedded in optimal cutting temperature medium (OCT) compound and then sectioned into 10 μm-thick sections using the Leica CX3050S cryostat. The generated sections were selected based on H&E staining with focusing on morphology and orientation. A further selection on samples was conducted based on the RNA integrity number obtained from Agilent2100 Bioanalyzer. Tissue optimization was performed respectively on tumor core and tumour-normal interface samples. After optimization, the Visium spatial gene expression protocol from 10X Genomics was performed using the Library Preparation slide and following the manufacturer’s protocol. After transcript capture, Visium Library Preparation was further performed following the manufacturer’s protocol. All images for this process were scanned at 40× on Hamamatsu NanoZoomer S60. cDNA libraries from five tumor core and 11 tumor normal interface samples were diluted and pooled to a final concentration of 2.25 nM (200 μL volume) and sequenced on 2× SP flow cells of Illumina NovaSeq 6000.

#### Visium data processing

Sequencing reads from 10x Genomics Visium libraries were aligned to the human transcriptome reference GRCh38-2020-A using 10x Genomics SpaceRanger (v.1.3.0) and exonic reads were used to produce mRNA count matrices for each sample. 10x Genomics SpaceRanger was also used to align paired histology images with mRNA capture spot positions in the Visium slide. We further integrated and processed SpaceRanger outputs using Scanpy (v.1.8.2). Following Scanpy pipeline, we filtered out Visium spots with the number of counts smaller than 2,000 and greater than 35,000, and the number of genes smaller than 500. Visium spots with a mitochondrion gene percentage greater than 20% were further filtered out. After quality check and data filtering, we removed two poor quality slides whose numbers of spots were smaller than 500 (6800STDY12499504 and 6800STDY12499505).

#### Spatial mapping of cell types with cell2location

To spatially map the cell types that we annotated in scRNA-seq data to spatial transcriptomic data, we applied cell2location to integrating scRNA-seq data with 10x Genomics Visium mRNA count matrices as described previously.^43^ In brief, the cell2location model estimates the abundance of each cell population in each location by decomposing mRNA counts in 10x Genomics Visium data using the transcriptional signatures of reference cell types. Two major steps were in analysis using cell2location: (1) We applied a negative binomial regression model implemented in cell2location and estimated the reference signature of cell types we annotated based on scRNA-seq data. In this step, we used an unnormalized mRNA count matrix as input and filtered it to 13,042 genes and 261,202 cells (cells that were annotated as unknown, low-quality and patient specific were removed from this analysis). Donor IDs were regarded as the batch category and the following parameters were used to train the model: ‘max_epochs’ = 120, ‘batch_size’ = 2500, ‘train_size’ = 1 and ‘Ir’ = 0.002. (2) The reference signature model was further used by cell2location to estimate spatial abundance of cell types. We kept genes that were shared with scRNA-seq and estimated the abundance of cell types in tumor core and interface groups respectively. In this step, cell2location was used with the following parameter settings: training iterations: 20,000, number of cells per location N = 20, ‘detection_alpha’ = 200. We plotted cells of interest (i.e., EMT and PT tumor cells) in each slide and excluded one slide (6800STDY12499409) where no tumor cells were clearly mapped in the spatial data.

We examined the localisation pattern between TR Mac.2 and different tumor cell subtypes. To take into account cell-types proximally co-localised, the 6 adjacent spots were identified for each spot in the slide. Where there were less than 6 surrounding spots (for spots on an edge of the tissue for example) or where one of the spots had already been used as another spots neighbor, the spot in question was skipped. This iterative grouping of spot and neighbor provided a comprehensive non-overlapping map of 7-spot units covering the whole slide. The mean of the computed cell2location abundance scores was computed for each of these 7 spot units and the Pearson correlation was calculated between these for each RCC program (defined as "TR Mac.2″, "EMT", "Stress", "MHCII", "PT", "CD"). Correlations of abundances between "TR Mac2″ and each other RCC program were selected from the resulting correlation matrix and plotted separately for interface and tumor core samples.

### Quantification and statistical analysis

All statistical analyses were performed using R (version 4.0.4). Two-sided Wilcoxon rank-sum test was applied to examining whether T cell clonotypes refine to a range of pseudotime values, whether TCR heterogeneity is more strongly correlated with spatial localisation or somatic heterogeneity, and whether certain TCGA subtype of tumors has significantly higher EMT score. Tukey test was used to investigate the relationship of TCR clonal expansion and tissue locations among different TCR categories. In *De Novo* mutation calling, binomial test was applied in each cell to test whether there is significantly higher reference than variant counts and was used to test whether called mutations show a significant strand bias. Fisher’s exact was used to test whether called mutations are significantly enriched by cell lineage. Descriptions of statistical tests performed for each individual analysis are provided in Figure legends and method details. No methods were used to determine whether the data met assumptions of the statistical approach.

## Data Availability

• The data generated by this paper is available through the following means: The genome sequence data reported in this paper is available at the European Genome-Phenome Archive: EGAD00001008029 for whole-exome sequencing data, EGAD00001008030 for the single cell RNA sequencing data, and EGAD00001008781 for the spatial transcriptomic data. Our single cell RNA sequencing and spatial transcriptomics data are available to download as h5ad objects in Mendeley Data: https://doi.org/10.17632/g67bkbnhhg.1. Our data can be explored on an online web portal https://www.sanger.ac.uk/project/microenvironment-of-kidney-cancer. Other data involved in this study were obtained from the following sources: TCGA ccRCC cohort (Cancer Genome Atlas Research^2^), Biet al^26^ (the provided Single Cell Portal), Krishna et al^27^ (SRZ: SRZ190804), Braun et al^28^ (supplementary materials), Borcherding et al^31^ (GEO: GSE121638) and Cheng et al^39^ (GEO: GSE154763). • Code and pipeline for deSCeRNAmut is available at Github: https://github.com/ThomasJamesMitchell/deSCeRNAMut. The code generated during this study is available at Github: https://github.com/ruoyan-li/RCC-spatial-mapping. Additional DOIs for code used in this study are listed in the key resources table. • Any additional information required to reanalyze the data reported in this work paper is available from the lead contact upon request.

## Supplemental information

Document S1. Figures S1–S7

Table S1. Clinicopathological information of patients in the cohort, related to Figure 1 and STAR Methods

Table S2. Information on scRNA-seq and Visium spatial transcriptomics, related to Figure 1

Table S3. Information on LCM samples and WES, related to Figure 1

Table S4. Top differentially expressed genes in the subclustering analysis of different cell lineages, related to Figure 1

Table S5. Gene list of intra-tumor expression programs and meta-programs, related to Figure 6

Document S2. Article plus supplemental information

## References

[1] Hsieh J.J., Purdue M.P., Signoretti S., Swanton C., Albiges L., Schmidinger M., Heng D.Y., Larkin J., Ficarra V. (2017). Renal cell carcinoma. Nat. Rev. Dis. Primers.

[2] Cancer Genome Atlas Research Network (2013). Comprehensive molecular characterization of clear cell renal cell carcinoma. Nature.

[3] Dalgliesh G.L., Furge K., Greenman C., Chen L., Bignell G., Butler A., Davies H., Edkins S., Hardy C., Latimer C. (2010). Systematic sequencing of renal carcinoma reveals inactivation of histone modifying genes. Nature.

[4] Mitchell T.J., Turajlic S., Rowan A., Nicol D., Farmery J.H.R., O'Brien T., Martincorena I., Tarpey P., Angelopoulos N., Yates L.R. (2018). Timing the landmark events in the evolution of clear cell renal cell cancer: TRACERx renal. Cell.

[5] Turajlic S., Xu H., Litchfield K., Rowan A., Chambers T., Lopez J.I., Nicol D., O'Brien T., Larkin J., Horswell S. (2018). Tracking cancer evolution reveals constrained routes to metastases: TRACERx renal. Cell.

[6] Varela I., Tarpey P., Raine K., Huang D., Ong C.K., Stephens P., Davies H., Jones D., Lin M.L., Teague J. (2011). Exome sequencing identifies frequent mutation of the SWI/SNF complex gene PBRM1 in renal carcinoma. Nature.

[7] Gerlinger M., Horswell S., Larkin J., Rowan A.J., Salm M.P., Varela I., Fisher R., McGranahan N., Matthews N., Santos C.R. (2014). Genomic architecture and evolution of clear cell renal cell carcinomas defined by multiregion sequencing. Nat. Genet..

[8] Gerlinger M., Rowan A.J., Horswell S., Math M., Larkin J., Endesfelder D., Gronroos E., Martinez P., Matthews N., Stewart A. (2012). Intratumor heterogeneity and branched evolution revealed by multiregion sequencing. N. Engl. J. Med..

[9] Şenbabaoğlu Y., Gejman R.S., Winer A.G., Liu M., Van Allen E.M., de Velasco G., Miao D., Ostrovnaya I., Drill E., Luna A. (2016). Tumor immune microenvironment characterization in clear cell renal cell carcinoma identifies prognostic and immunotherapeutically relevant messenger RNA signatures. Genome Biol..

[10] Yoshihara K., Shahmoradgoli M., Martínez E., Vegesna R., Kim H., Torres-Garcia W., Treviño V., Shen H., Laird P.W., Levine D.A. (2013). Inferring tumour purity and stromal and immune cell admixture from expression data. Nat. Commun..

[11] Motzer R.J., Escudier B., McDermott D.F., George S., Hammers H.J., Srinivas S., Tykodi S.S., Sosman J.A., Procopio G., Plimack E.R. (2015). Nivolumab versus everolimus in advanced renal-cell carcinoma. N. Engl. J. Med..

[12] Rini B.I., Plimack E.R., Stus V., Gafanov R., Hawkins R., Nosov D., Pouliot F., Alekseev B., Soulières D., Melichar B. (2019). Pembrolizumab plus axitinib versus sunitinib for advanced renal-cell carcinoma. N. Engl. J. Med..

[13] Clark D.J., Dhanasekaran S.M., Petralia F., Pan J., Song X., Hu Y., da Veiga Leprevost F., Reva B., Lih T.S.M., Chang H.Y. (2019). Integrated proteogenomic characterization of clear cell renal cell carcinoma. Cell.

[14] Chevrier S., Levine J.H., Zanotelli V.R.T., Silina K., Schulz D., Bacac M., Ries C.H., Ailles L., Jewett M.A.S., Moch H. (2017). An immune atlas of clear cell renal cell carcinoma. Cell.

[15] Jerby-Arnon L., Shah P., Cuoco M.S., Rodman C., Su M.J., Melms J.C., Leeson R., Kanodia A., Mei S., Lin J.R. (2018). A cancer cell program promotes T cell exclusion and resistance to checkpoint blockade. Cell.

[16] Puram S.V., Tirosh I., Parikh A.S., Patel A.P., Yizhak K., Gillespie S., Rodman C., Luo C.L., Mroz E.A., Emerick K.S. (2017). Single-cell transcriptomic analysis of primary and metastatic tumor ecosystems in head and neck cancer. Cell.

[17] Tirosh I., Izar B., Prakadan S.M., Wadsworth M.H., Treacy D., Trombetta J.J., Rotem A., Rodman C., Lian C., Murphy G. (2016). Dissecting the multicellular ecosystem of metastatic melanoma by single-cell RNA-seq. Science.

[18] Azizi E., Carr A.J., Plitas G., Cornish A.E., Konopacki C., Prabhakaran S., Nainys J., Wu K., Kiseliovas V., Setty M. (2018). Single-cell map of diverse immune phenotypes in the breast tumor microenvironment. Cell.

[19] Li H., van der Leun A.M., Yofe I., Lubling Y., Gelbard-Solodkin D., van Akkooi A.C.J., van den Braber M., Rozeman E.A., Haanen J.B.A.G., Blank C.U. (2019). Dysfunctional CD8 T cells form a proliferative, dynamically regulated compartment within human melanoma. Cell.

[20] Zhang L., Li Z., Skrzypczynska K.M., Fang Q., Zhang W., O'Brien S.A., He Y., Wang L., Zhang Q., Kim A. (2020). Single-cell analyses inform mechanisms of myeloid-targeted therapies in colon cancer. Cell.

[21] Ji A.L., Rubin A.J., Thrane K., Jiang S., Reynolds D.L., Meyers R.M., Guo M.G., George B.M., Mollbrink A., Bergenstråhle J. (2020). Multimodal analysis of composition and spatial architecture in human squamous cell carcinoma. Cell.

[22] Sharma A., Seow J.J.W., Dutertre C.A., Pai R., Blériot C., Mishra A., Wong R.M.M., Singh G.S.N., Sudhagar S., Khalilnezhad S. (2020). Onco-fetal reprogramming of endothelial cells drives immunosuppressive macrophages in hepatocellular carcinoma. Cell.

[23] Davidson S., Efremova M., Riedel A., Mahata B., Pramanik J., Huuhtanen J., Kar G., Vento-Tormo R., Hagai T., Chen X. (2020). Single-cell RNA sequencing reveals a dynamic stromal niche that supports tumor growth. Cell Rep..

[24] Hornburg M., Desbois M., Lu S., Guan Y., Lo A.A., Kaufman S., Elrod A., Lotstein A., DesRochers T.M., Munoz-Rodriguez J.L. (2021). Single-cell dissection of cellular components and interactions shaping the tumor immune phenotypes in ovarian cancer. Cancer Cell.

[25] Young M.D., Mitchell T.J., Vieira Braga F.A., Tran M.G.B., Stewart B.J., Ferdinand J.R., Collord G., Botting R.A., Popescu D.M., Loudon K.W. (2018). Single-cell transcriptomes from human kidneys reveal the cellular identity of renal tumors. Science.

[26] Bi K., He M.X., Bakouny Z., Kanodia A., Napolitano S., Wu J., Grimaldi G., Braun D.A., Cuoco M.S., Mayorga A. (2021). Tumor and immune reprogramming during immunotherapy in advanced renal cell carcinoma. Cancer Cell.

[27] Krishna C., DiNatale R.G., Kuo F., Srivastava R.M., Vuong L., Chowell D., Gupta S., Vanderbilt C., Purohit T.A., Liu M. (2021). Single-cell sequencing links multiregional immune landscapes and tissue-resident T cells in ccRCC to tumor topology and therapy efficacy. Cancer Cell.

[28] Braun D.A., Street K., Burke K.P., Cookmeyer D.L., Denize T., Pedersen C.B., Gohil S.H., Schindler N., Pomerance L., Hirsch L. (2021). Progressive immune dysfunction with advancing disease stage in renal cell carcinoma. Cancer Cell.

[29] Minervini A., Campi R., Di Maida F., Mari A., Montagnani I., Tellini R., Tuccio A., Siena G., Vittori G., Lapini A. (2018). Tumor-parenchyma interface and long-term oncologic outcomes after robotic tumor enucleation for sporadic renal cell carcinoma. Urol. Oncol..

[30] Sanchez A., Furberg H., Kuo F., Vuong L., Ged Y., Patil S., Ostrovnaya I., Petruzella S., Reising A., Patel P. (2020). Transcriptomic signatures related to the obesity paradox in patients with clear cell renal cell carcinoma: a cohort study. Lancet Oncol..

[31] Borcherding N., Vishwakarma A., Voigt A.P., Bellizzi A., Kaplan J., Nepple K., Salem A.K., Jenkins R.W., Zakharia Y., Zhang W. (2021). Mapping the immune environment in clear cell renal carcinoma by single-cell genomics. Commun. Biol..

[32] Stewart B.J., Ferdinand J.R., Young M.D., Mitchell T.J., Loudon K.W., Riding A.M., Richoz N., Frazer G.L., Staniforth J.U.L., Vieira Braga F.A. (2019). Spatiotemporal immune zonation of the human kidney. Science.

[33] Workel H.H., Lubbers J.M., Arnold R., Prins T.M., van der Vlies P., de Lange K., Bosse T., van Gool I.C., Eggink F.A., Wouters M.C.A. (2019). A transcriptionally distinct CXCL13(+)CD103(+)CD8(+) T-cell population is associated with B-cell recruitment and neoantigen load in human cancer. Cancer Immunol. Res..

[34] Jamal-Hanjani M., Wilson G.A., McGranahan N., Birkbak N.J., Watkins T.B.K., Veeriah S., Shafi S., Johnson D.H., Mitter R., Rosenthal R. (2017). Tracking the evolution of non-small-cell lung cancer. N. Engl. J. Med..

[35] Huang H., Wang C., Rubelt F., Scriba T.J., Davis M.M. (2020). Analyzing the Mycobacterium tuberculosis immune response by T-cell receptor clustering with GLIPH2 and genome-wide antigen screening. Nat. Biotechnol..

[36] Mitchell E., Spencer Chapman M., Williams N., Dawson K.J., Mende N., Calderbank E.F., Jung H., Mitchell T., Coorens T.H.H., Spencer D.H. (2022). Clonal dynamics of haematopoiesis across the human lifespan. Nature.

[37] Kar S.P., Quiros P.M., Gu M., Jiang T., Mitchell J., Langdon R., Iyer V., Barcena C., Vijayabaskar M.S., Fabre M.A. (2022). Genome-wide analyses of 200, 453 individuals yield new insights into the causes and consequences of clonal hematopoiesis. Nat. Genet..

[38] Welch J.S., Ley T.J., Link D.C., Miller C.A., Larson D.E., Koboldt D.C., Wartman L.D., Lamprecht T.L., Liu F., Xia J. (2012). The origin and evolution of mutations in acute myeloid leukemia. Cell.

[39] Cheng S., Li Z., Gao R., Xing B., Gao Y., Yang Y., Qin S., Zhang L., Ouyang H., Du P. (2021). A pan-cancer single-cell transcriptional atlas of tumor infiltrating myeloid cells. Cell.

[40] Jaitin D.A., Adlung L., Thaiss C.A., Weiner A., Li B., Descamps H., Lundgren P., Bleriot C., Liu Z., Deczkowska A. (2019). Lipid-associated macrophages control metabolic homeostasis in a trem2-dependent manner. Cell.

[41] Lavin Y., Kobayashi S., Leader A., Amir E.A.D., Elefant N., Bigenwald C., Remark R., Sweeney R., Becker C.D., Levine J.H. (2017). Innate immune landscape in early lung adenocarcinoma by paired single-cell analyses. Cell.

[42] Liu F., Dai S., Feng D., Qin Z., Peng X., Sakamuri S.S.V.P., Ren M., Huang L., Cheng M., Mohammad K.E. (2020). Distinct fate, dynamics and niches of renal macrophages of bone marrow or embryonic origins. Nat. Commun..

[43] Kleshchevnikov V., Shmatko A., Dann E., Aivazidis A., King H.W., Li T., Elmentaite R., Lomakin A., Kedlian V., Gayoso A. (2022). Cell2location maps fine-grained cell types in spatial transcriptomics. Nat. Biotechnol..

[44] Brooks S.A., Brannon A.R., Parker J.S., Fisher J.C., Sen O., Kattan M.W., Hakimi A.A., Hsieh J.J., Choueiri T.K., Tamboli P. (2014). ClearCode34: a prognostic risk predictor for localized clear cell renal cell carcinoma. Eur. Urol..

[45] Motzer R.J., Banchereau R., Hamidi H., Powles T., McDermott D., Atkins M.B., Escudier B., Liu L.F., Leng N., Abbas A.R. (2020). Molecular subsets in renal cancer determine outcome to checkpoint and angiogenesis blockade. Cancer Cell.

[46] Rini B., Goddard A., Knezevic D., Maddala T., Zhou M., Aydin H., Campbell S., Elson P., Koscielny S., Lopatin M. (2015). A 16-gene assay to predict recurrence after surgery in localised renal cell carcinoma: development and validation studies. Lancet Oncol..

[47] Browaeys R., Saelens W., Saeys Y. (2020). NicheNet: modeling intercellular communication by linking ligands to target genes. Nat. Methods.

[48] Petrella B.L., Vincenti M.P. (2012). Interleukin-1beta mediates metalloproteinase-dependent renal cell carcinoma tumor cell invasion through the activation of CCAAT enhancer binding protein beta. Cancer Med..

[49] Oliveira G., Stromhaug K., Klaeger S., Kula T., Frederick D.T., Le P.M., Forman J., Huang T., Li S., Zhang W. (2021). Phenotype, specificity and avidity of antitumour CD8(+) T cells in melanoma. Nature.

[50] Beshnova D., Ye J., Onabolu O., Moon B., Zheng W., Fu Y.X., Brugarolas J., Lea J., Li B. (2020). De novo prediction of cancer-associated T cell receptors for noninvasive cancer detection. Sci. Transl. Med..

[51] Smith C.G., Moser T., Mouliere F., Field-Rayner J., Eldridge M., Riediger A.L., Chandrananda D., Heider K., Wan J.C.M., Warren A.Y. (2020). Comprehensive characterization of cell-free tumor DNA in plasma and urine of patients with renal tumors. Genome Med..

[52] Chittezhath M., Dhillon M.K., Lim J.Y., Laoui D., Shalova I.N., Teo Y.L., Chen J., Kamaraj R., Raman L., Lum J. (2014). Molecular profiling reveals a tumor-promoting phenotype of monocytes and macrophages in human cancer progression. Immunity.

[53] Aggen D.H., Ager C.R., Obradovic A.Z., Chowdhury N., Ghasemzadeh A., Mao W., Chaimowitz M.G., Lopez-Bujanda Z.A., Spina C.S., Hawley J.E. (2021). Blocking IL1 beta promotes tumor regression and remodeling of the myeloid compartment in a renal cell carcinoma model: multidimensional analyses. Clin. Cancer Res..

[54] Ridker P.M., MacFadyen J.G., Thuren T., Everett B.M., Libby P., Glynn R.J., CANTOS Trial Group (2017). Effect of interleukin-1beta inhibition with canakinumab on incident lung cancer in patients with atherosclerosis: exploratory results from a randomised, double-blind, placebo-controlled trial. Lancet.

[55] Domínguez Conde C., Xu C., Jarvis L.B., Rainbow D.B., Wells S.B., Gomes T., Howlett S.K., Suchanek O., Polanski K., King H.W. (2022). Cross-tissue immune cell analysis reveals tissue-specific features in humans. Science.

[56] Stuart T., Butler A., Hoffman P., Hafemeister C., Papalexi E., Mauck W.M., Hao Y., Stoeckius M., Smibert P., Satija R. (2019). Comprehensive integration of single-cell data. Cell.

[57] Wang K., Li M., Hakonarson H. (2010). ANNOVAR: functional annotation of genetic variants from high-throughput sequencing data. Nucleic Acids Res..

[58] Li H., Handsaker B., Wysoker A., Fennell T., Ruan J., Homer N., Marth G., Abecasis G., Durbin R., 1000 Genome Project Data Processing Subgroup (2009). The sequence alignment/map format and SAMtools. Bioinformatics.

[59] Young M.D., Behjati S. (2020). SoupX removes ambient RNA contamination from droplet-based single-cell RNA sequencing data. GigaScience.

[60] McGinnis C.S., Murrow L.M., Gartner Z.J. (2019). DoubletFinder: doublet detection in single-cell RNA sequencing data using artificial nearest neighbors. Cell Syst..

[61] Ellis P., Moore L., Sanders M.A., Butler T.M., Brunner S.F., Lee-Six H., Osborne R., Farr B., Coorens T.H.H., Lawson A.R.J. (2021). Reliable detection of somatic mutations in solid tissues by laser-capture microdissection and low-input DNA sequencing. Nat. Protoc..

[62] Li H., Durbin R. (2009). Fast and accurate short read alignment with Burrows-Wheeler transform. Bioinformatics.

[63] Coorens T.H.H., Treger T.D., Al-Saadi R., Moore L., Tran M.G.B., Mitchell T.J., Tugnait S., Thevanesan C., Young M.D., Oliver T.R.W. (2019). Embryonal precursors of Wilms tumor. Science.

[64] Van Loo P., Nordgard S.H., Lingjærde O.C., Russnes H.G., Rye I.H., Sun W., Weigman V.J., Marynen P., Zetterberg A., Naume B. (2010). Allele-specific copy number analysis of tumors. Proc. Natl. Acad. Sci. USA.

[65] Bolli N., Avet-Loiseau H., Wedge D.C., Van Loo P., Alexandrov L.B., Martincorena I., Dawson K.J., Iorio F., Nik-Zainal S., Bignell G.R. (2014). Heterogeneity of genomic evolution and mutational profiles in multiple myeloma. Nat. Commun..

[66] Liu F., Zhang Y., Zhang L., Li Z., Fang Q., Gao R., Zhang Z. (2019). Systematic comparative analysis of single-nucleotide variant detection methods from single-cell RNA sequencing data. Genome Biol..

[67] Condamine T., Dominguez G.A., Youn J.I., Kossenkov A.V., Mony S., Alicea-Torres K., Tcyganov E., Hashimoto A., Nefedova Y., Lin C. (2016). Lectin-type oxidized LDL receptor-1 distinguishes population of human polymorphonuclear myeloid-derived suppressor cells in cancer patients. Sci. Immunol..

[68] La Manno G., Soldatov R., Zeisel A., Braun E., Hochgerner H., Petukhov V., Lidschreiber K., Kastriti M.E., Lönnerberg P., Furlan A. (2018). RNA velocity of single cells. Nature.

[69] Bergen V., Lange M., Peidli S., Wolf F.A., Theis F.J. (2020). Generalizing RNA velocity to transient cell states through dynamical modeling. Nat. Biotechnol..

