# Supplementary material for: Mapping single-cell transcriptomes in the intra-tumoral and associated territories of kidney cancer
Source: Cancer Cell. 2022 Dec 12;40(12):1583–1599.e10. doi: 10.1016/j.ccell.2022.11.001 (PMC9767677; doi:10.1016/j.ccell.2022.11.001)
Supplement: Document S1. Figures S1–S7 [file mmc1.pdf]

Cancer Cell, Volume *40*

Supplemental information

Mapping single-cell transcriptomes in the intra-tumoral and associated territories of kidney cancer

Ruoyan Li, John R. Ferdinand, Kevin W. Loudon, Georgina S. Bowyer, Sean Laidlaw, Francesc Muyas, Lira Mamanova, Joana B. Neves, Liam Bolt, Eirini S. Fasouli, Andrew R.J. Lawson, Matthew D. Young, Yvette Hooks, Thomas R.W. Oliver, Timothy M. Butler, James N. Armitage, Tev Aho, Antony C.P. Riddick, Vincent Gnanapragasam, Sarah J. Welsh, Kerstin B. Meyer, Anne Y. Warren, Maxine G.B. Tran, Grant D. Stewart, Isidro Cortés-Ciriano, Sam Behjati, Menna R. Clatworthy, Peter J. Campbell, Sarah A. Teichmann, and Thomas J. Mitchell

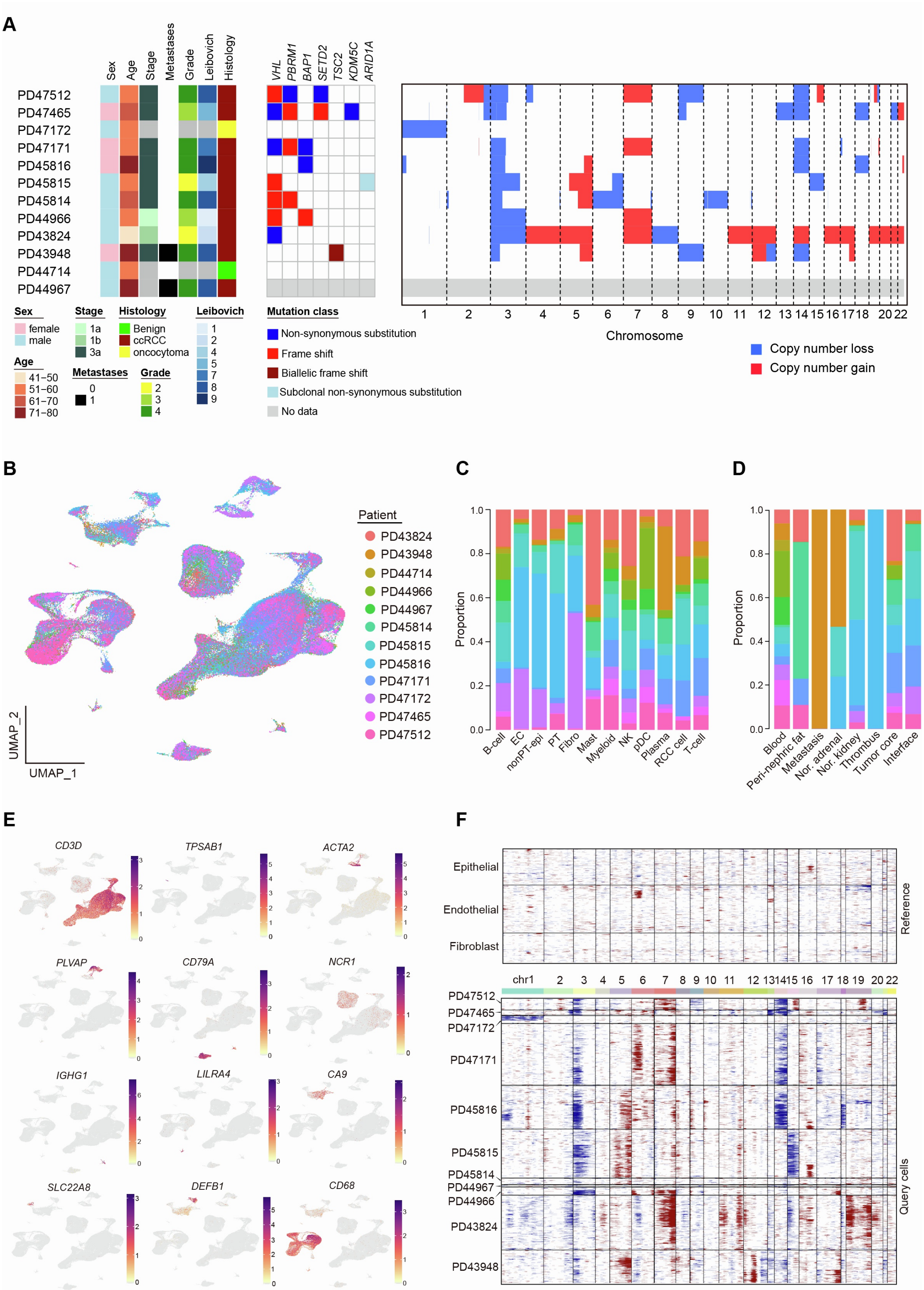

Figure S1. Basic information of the study cohort and the data, related to Figure 1.
(A) Heatmap illustrating the clinical features (left panel) and the genomic landscape (middle panel) and copy number profiles (right panel) of the tumors sequenced. (B) UMAP, (C) cell type, and (D) sampled region depicting the interpatient variability of scRNA-seq results. (E) UMAP showing marker gene expression for all cells. (F) Copy number inference based on scRNA-seq data.

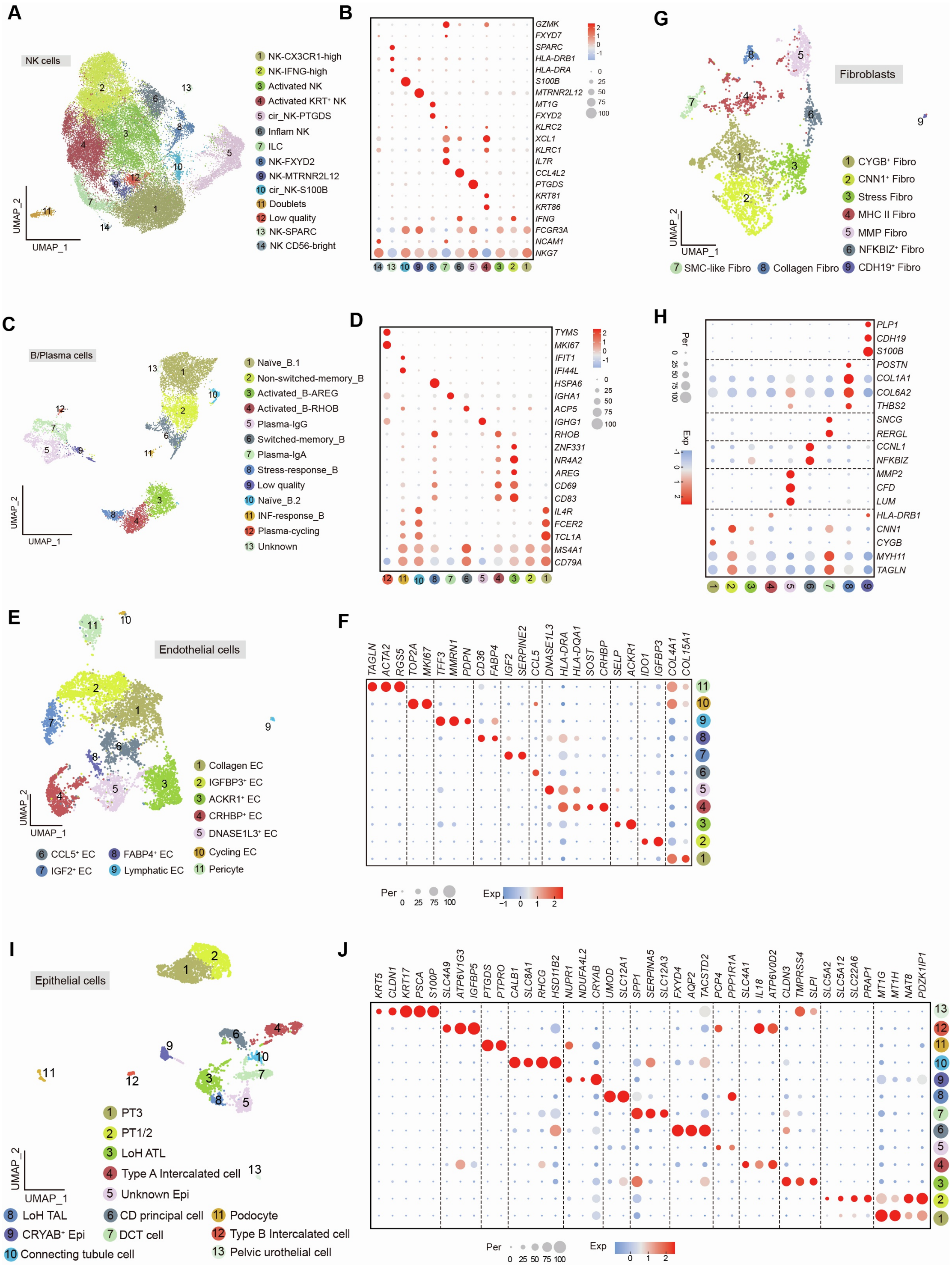

Figure S2. Sub-clustering of NK, B cell, stromal and epithelial cell compartments, related to Figure 1.

(A, C, E, G and I) UMAPs showing sub-clustering and annotations of NK, B/Plasma, endothelial, fibroblast and epithelial cell compartments. (B, D, F, H and J) Dot plots showing differentially expressed genes in each cluster from the sub-clustering analysis of different cell compartments. Dot size represents the percentage of cells and color represents expression level.

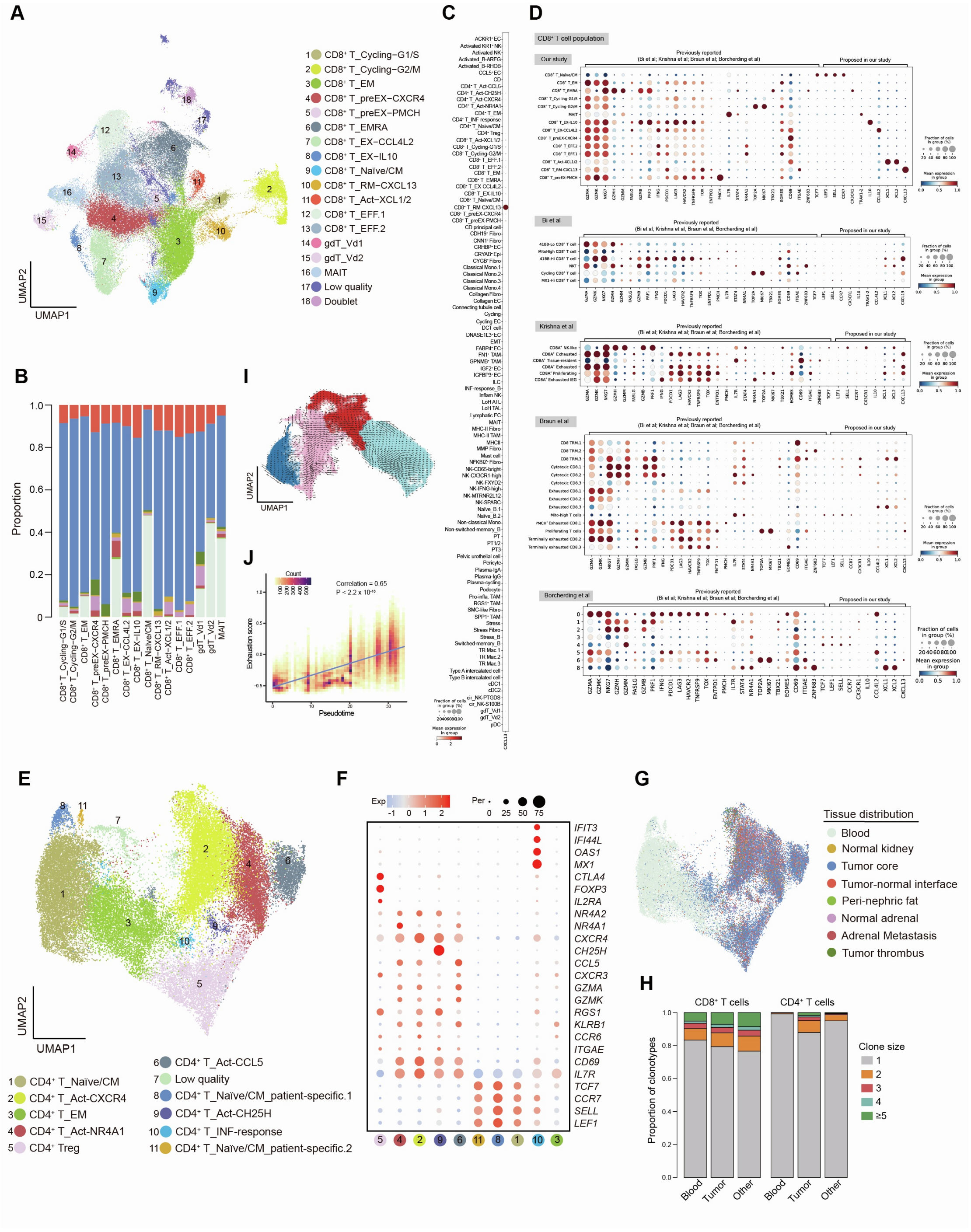

Figure S3. Spatial and transcriptomic heterogeneity of T cell compartment, related to Figure 3.

(A) UMAP of the sub-clustering result. (B) Bar plot showing tissue distribution of T cell subsets. (C) Dot plot showing the expression of *CXCL13* among all cell types. (D) Cross-study comparison of potential marker genes in CD8^{+} T cell compartment. (E) UMAP of the sub-clustering result. (F) Dot plot showing marker gene expression, and (G) UMAP of tissue distribution of CD4^{+} T cells. (H) Bar plot showing the comparison of TCR clonal expansion between CD8^{+} and CD4^{+} T cells, breaking down into three different locations: blood, tumor and other regions. (I) UMAP showing the RNA velocity result. (J) Exhaustion score across the pseudotime trajectory of CD8^{+} T cells. Statistical analysis by Pearson correlation test.

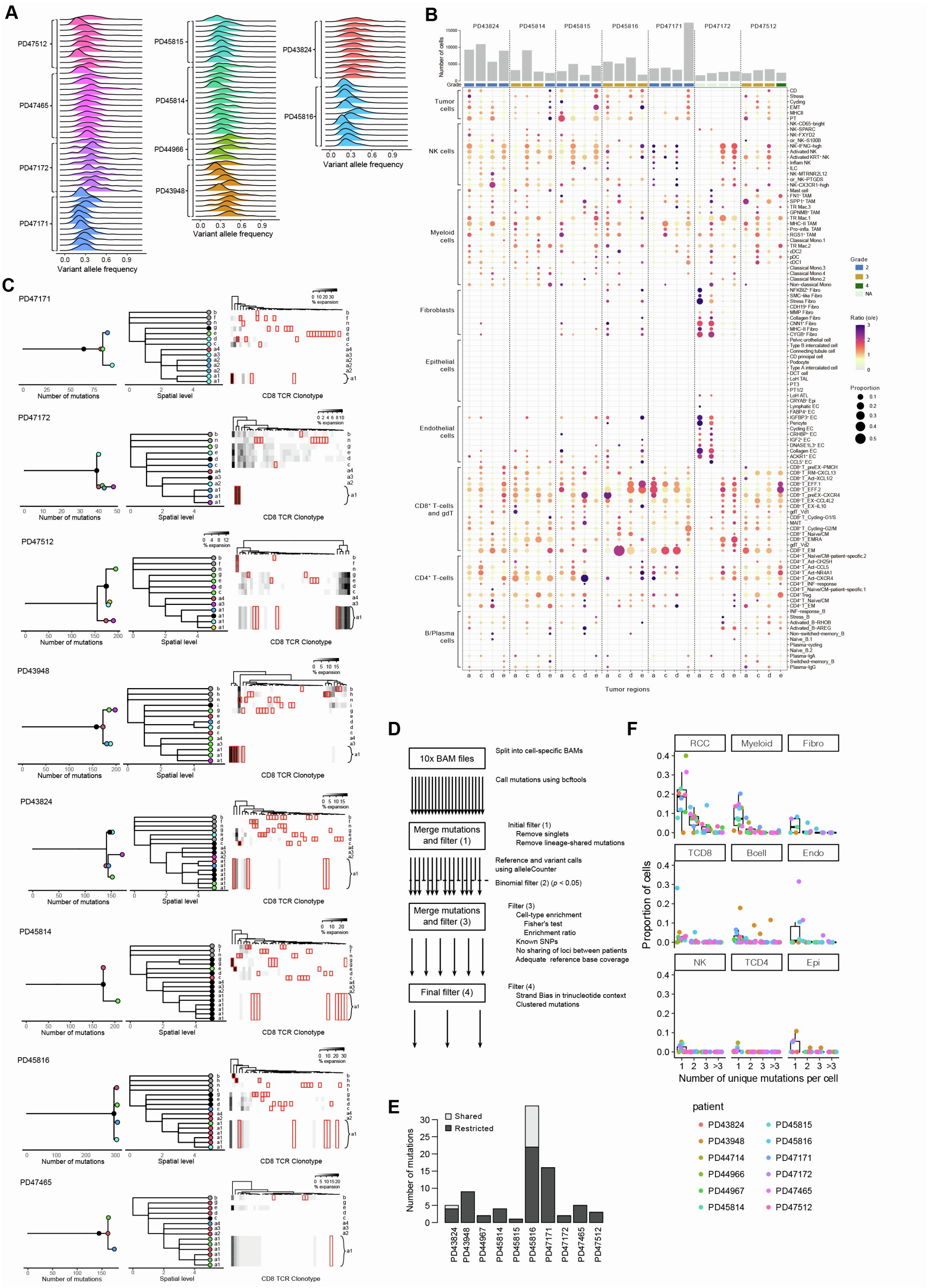

Figure S4. Somatic mutation analysis and heterogeneity of TME and TCR, related to Figure 4. (A) Density plots showing the VAF distribution of mutations called from WES data from LCM biopsies of tumor samples. (B) Dot plots showing heterogeneity of different tumor regions. We excluded the benign thick-walled cyst case (PD44714) and three cases (PD47465, PD44966 and PD43948) in which only one tumor region has scRNA-seq data. (C) Comparison of WES derived phylogenies (left) with geographic location (center) and CD8^{+} TCR clonotype expansion (right) for each patient with matching data. Colors reference somatic clones to

spatial localization. Each column in the right panel represents a TCR clonotype, those with significant regional enrichment are highlighted in red. a, c, d, and e represents four different regions of the tumor core; g, tumor-normal interface; f, perinephric fat; n, normal kidney; b, peripheral blood; h, normal adrenal gland; i, adrenal metastasis; t, thrombus. (D) Schematic of the *de novo* mutation calling framework. (E) Bar chart showing benchmarking results of the number of called mutations in $CD8^+$ T cells derived from scRNA-seq data that are restricted to individual TCR clonotypes. (F) Comparison of the proportion of cells with one, two, three, and more than three mutations across the major cell types.

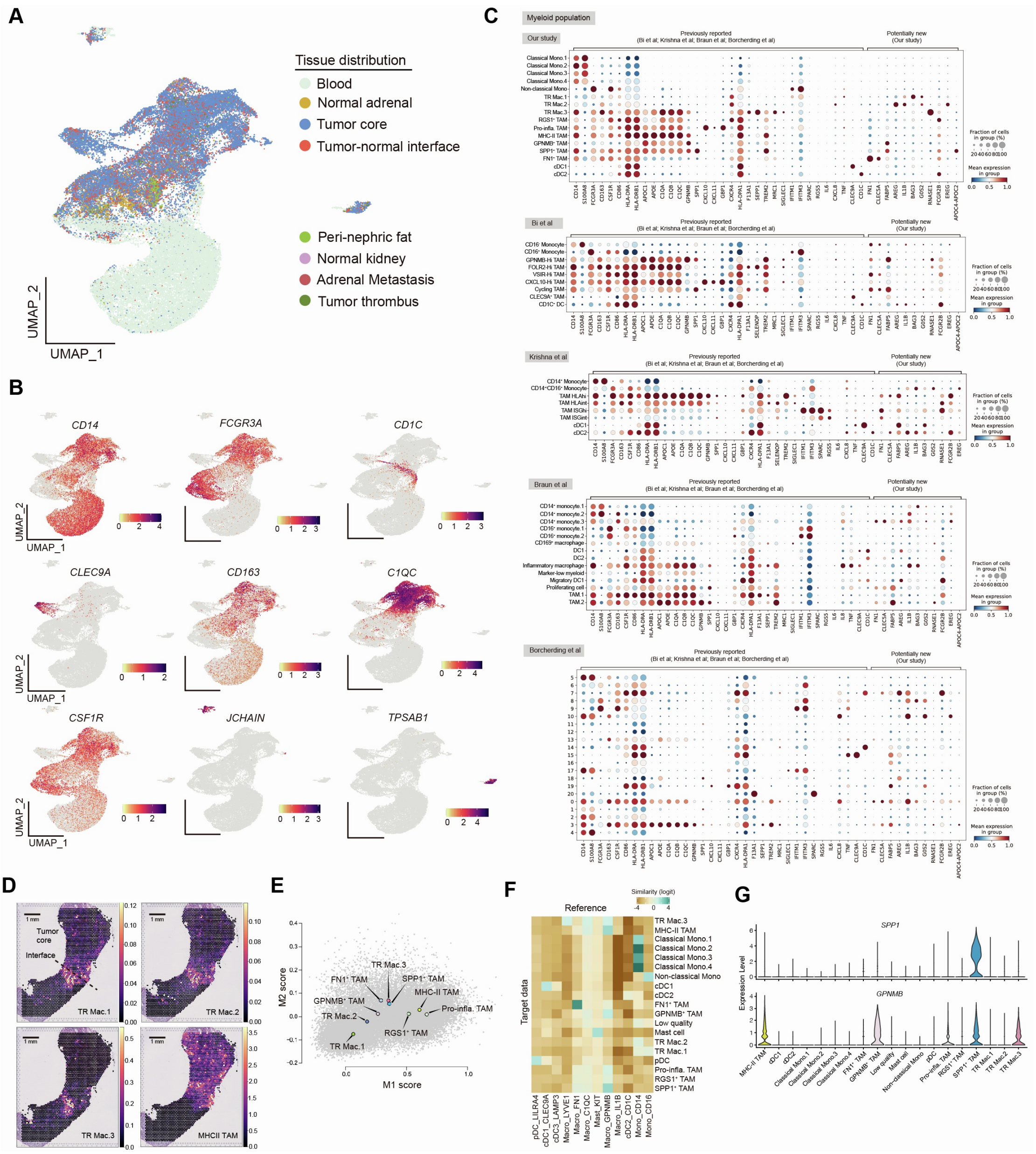

Figure S5. Sub-clustering analysis of myeloid cell compartment, related to Figure 5

(A) UMAP depicting tissue distribution of myeloid cells. (B) UMAPs showing marker gene expression in myeloid cells. (C) Cross-study comparison of potential marker genes in myeloid cell compartment (D) Spatial mapping of different macrophage subsets in a representative tumor-normal interface sample (PD45816) using cell2location. Estimated abundance (color intensity) is overlaid on a histology image. Scale bar, 1 mm. (E) Scatter plot of M1 versus M2 polarization for all of the macrophages showing individual cells (grey), and mean values for each subtype (colored). (F) Heatmap plotting the similarity of myeloid clusters derived in our study with those in a previous study (Cheng et al[39]). (G) Violin plots showing expression levels of *SPP1* and *GPNMB* across all myeloid cell subtypes. Data are presented as distributions.

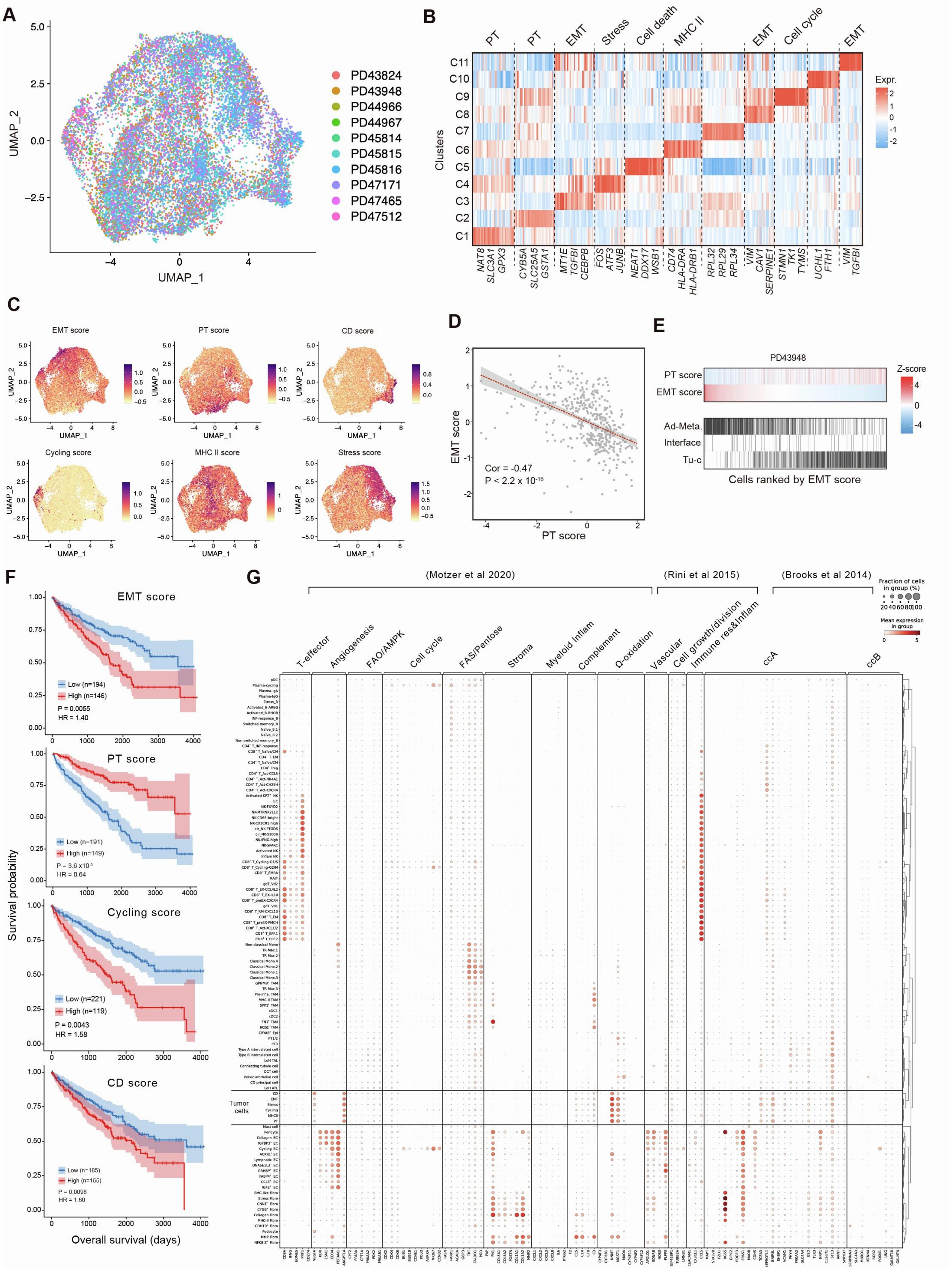

Figure S6. Tumor cell expression programs, related to Figure 6.

(A) UMAP showing the patients that RCC cells derived from. (B) Heatmap depicting the top DEG expression and assignment to meta-programs for RCC cell clusters. (C) UMAPs showing the relative expression of each meta-program for all RCC cells. (D) Correlation of EMT versus PT scores from bulk RNA sequencing data of the TCGA. Statistical analysis by Pearson correlation test. (E) Cells from patient donors PD43948, ranked by decreasing EMT score with corresponding PT score and cell location. Tu-c, tumor core. (F) Survival probability of patients according to stratification of bulk RNA sequencing from TCGA with single-cell derived meta-

programs. HR, hazard ratio. Statistical analysis by the Cox proportional hazards regression model. (G) Dot plot showing expression of clinical related signature genes reported previously among all cell types identified in our study.

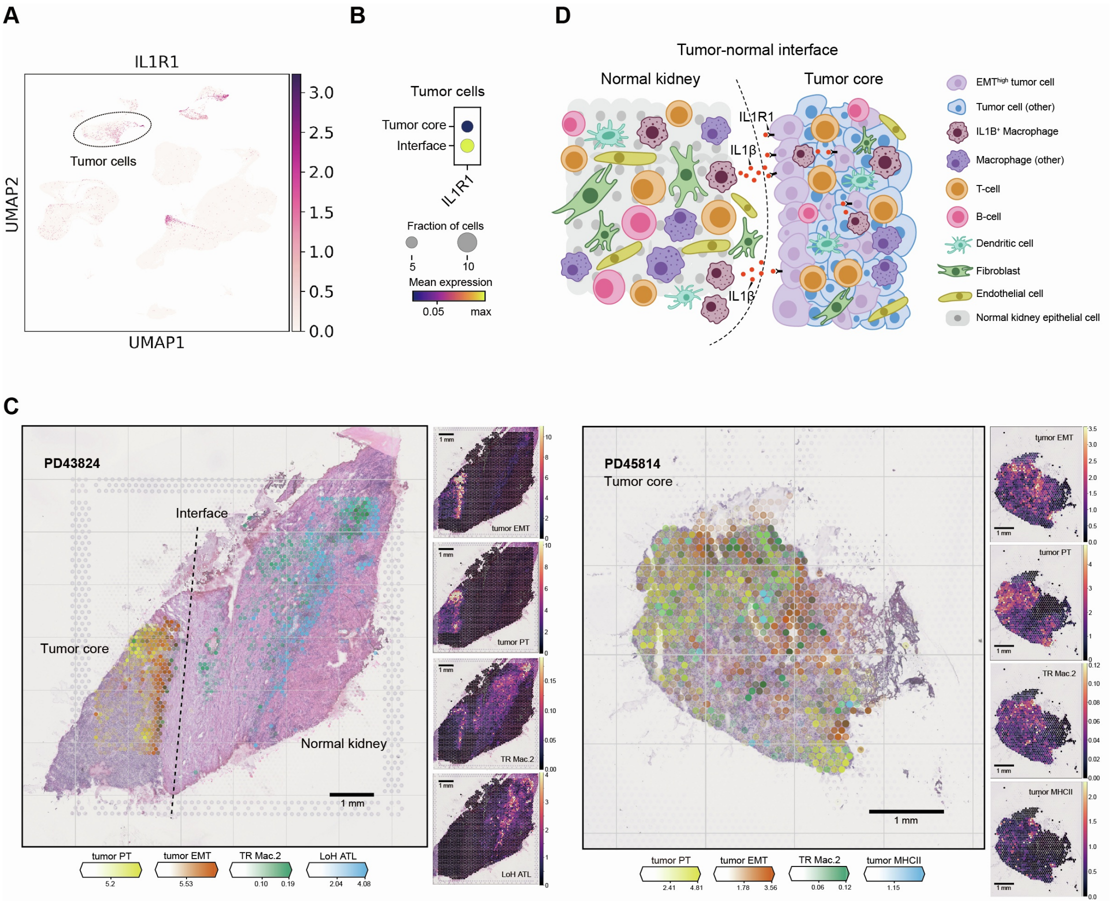

Figure S7. Cell-cell interactions in the ccRCC micro-environment, related to Figure 7.

(A) UMAP showing the expression of IL1β receptor gene *IL1R1*. Tumor cell population is highlighted. (B) Dot plot showing the expression of IL1β receptor gene *IL1R1* in tumor cells from the tumor core and tumor-normal interface. (C) Spatial mapping of EMT tumor cells, PT tumor cells and TR Mac.2 in Visium data for representative tumor-normal interface (PD43824) and tumor core (PD45814) using cell2location. Estimated abundance for cell types (color intensity) across locations (dots) is overlaid on histology images. Scale bar, 1 mm. (D) Schematic illustrating the importance of IL1β signaling between IL1B⁺ macrophages and RCC cells in promoting EMT.
